# Vitamin D – a systematic literature review for the 5th edition of the Nordic Nutrition Recommendations

**DOI:** 10.3402/fnr.v57i0.22671

**Published:** 2013-10-03

**Authors:** Christel Lamberg-Allardt, Magritt Brustad, Haakon E. Meyer, Laufey Steingrimsdottir

**Affiliations:** 1 Department of Food and Environmental Sciences, University of Helsinki, Helsinki, Finland; 2 UIT-The Arctic University of Norway, Tromsø, Norway; 3 Department of Community Medicine, Institute of Health and Society, University of Oslo, Oslo, Norway; 4 Norwegian Institute of Public Health, Oslo, Norway; 5 Unit for Nutrition Research, Landspitali University Hospital & University of Iceland, Reykjavik, Iceland

**Keywords:** vitamin D, vitamin D requirements, systematic review, bone health, health outcomes

## Abstract

**Background:**

The present literature review is part of the NNR5 project with the aim of reviewing and updating the scientific basis of the 4th edition of the Nordic Nutrition Recommendations (NNR) issued in 2004.

**Objectives:**

The overall aim was to review recent scientific data on the requirements and health effects of vitamin D and to report it to the NNR5 Working Group, who is responsible for updating the current dietary reference values valid in the Nordic countries.

**Methods:**

The electronic databases MEDLINE and Swemed were searched. We formulated eight questions which were used for the search. The search terms related to vitamin D status and intake and different health outcomes as well as to the effect of different vitamin D sources on vitamin D status. The search was done in two batches, the first covering January 2000–March 2010 and the second March 2009–February 2011. In the first search, we focused only on systematic literature reviews (SLRs) and in the second on SLRs and randomized control trials (RCTs) published after March 2009. Furthermore, we used snowballing for SLRs and IRCTs published between February 2011 and May 2012. The abstracts as well as the selected full-text papers were evaluated in pairs.

**Results:**

We found 1,706 studies in the two searches of which 28 studies were included in our review. We found 7 more by snowballing, thus 35 papers were included in total. Of these studies, 31 were SLRs and 4 were RCTs. The SLRs were generally of good or fair quality, whereas that of the included studies varied from good to poor. The heterogeneity of the studies included in the SLRs was large which made it difficult to interpret the results and provide single summary statements. One factor increasing the heterogeneity is the large variation in the assays used for assessing 25-hydroxyvitamin D concentration [25(OH)D], the marker of vitamin D status. The SLRs we have reviewed conclude that the evidence for a protective effect of vitamin D is only conclusive concerning bone health, total mortality and the risk of falling. Moreover, the effect was often only seen in persons with low basal 25(OH)D concentrations. In addition, most intervention studies leading to these conclusions report that intervention with vitamin D combined with calcium and not vitamin D alone gives these benefits. It was difficult to establish an optimal 25(OH)D concentration or vitamin D intake based on the SLRs, but there are evidence that a concentration of ≥50 nmol/l could be optimal. The dose–response studies relating vitamin D intake (fortification and supplementation) to S-25(OH)D suggested that an intake of 1–2.5 µg/day will increase the serum concentration by 1–2 nmol/l but this is dependent on the basal concentration with a response being greater when the basal concentration is low.

**Conclusion:**

Data show that a S-25(OH)D concentration of 50 nmol/l would reflect a sufficient vitamin D status. Results from this review support that the recommendation in NNR 2004 needs to be re-evaluated and increased for all age groups beyond 2 years of age. We refer to the total intake from food as well as supplements, given minimal sun exposure. Limited sunshine, however, does not reflect the situation for the majority of the Nordic population in the summertime. It should also be emphasized that there are large differences in results depending on assay methods and laboratories measuring 25(OH)D, adding to the uncertainty of determining an appropriate target concentration. Moreover, the dose–response of vitamin D on serum 25(OH)D-concentrations is not well established and is dependent on the basal concentrations, sunshine exposure and dietary intake. We advise that these uncertainties should be taken into account when setting the final Nordic recommendations.

This literature review is part of the NNR5 project with the aim of reviewing and updating the scientific basis of the 4th edition of the Nordic Nutrition Recommendations (NNR) issued in 2004 (1). The NNR5 project is mainly focused on a revision of those areas in which new scientific knowledge has emerged since the 4th edition with special relevance for the Nordic setting. A number of systematic literature reviews (SLRs) will form the basis for the establishment of dietary reference values in the 5th edition of NNR.

The dietary reference values for vitamin D in the 4th edition of the NNR are 10 µg/day for the age group 6–23 months, 7.5 µg/day for 2–60 years, 10 µg for 61 years and older, and for pregnant and lactating women 10 µg/day. The upper level of vitamin D intake for adults is 50 µg/day (1).

## Aims

The overall aim was to review recent scientific data on requirements and health effects of vitamin D and to report it to the NNR5 Working Group, who is responsible for updating the current dietary reference values valid in the Nordic countries. The SLR followed the guidelines for conducting systematic reviews set by the working group (2).

The specific objectives of the review on health effects of vitamin D in human nutrition were to:

1. review the scientific evidence to determine, based on a set of agreed criteria, dietary reference values for vitamin D for different life stages (infants, children, adolescents, adults, elderly and during pregnancy and lactation),
2. assess the requirement for adequate growth, development and maintenance of health of vitamin D,
3. assess the health effects of different intakes/exposures of vitamin D.

### Scientific background

In humans, vitamin D is obtained from the diet and through cutaneous synthesis in the presence of ultra-violet irradiation supplied by sunlight. Vitamin D is converted to 25-hydroxy-vitamin D [25(OH)D] in the liver and is transported in the circulation by a vitamin-D-binding protein, DBP (also named Gc-protein or Gc-globulin). The 25(OH)D concentration measured in serum or plasma is considered to be the best marker of vitamin D status.

The biologically active form, 1,25-dihydroxy-vitamin D [1,25-(OH)_2_-D], is formed in the kidneys from 25(OH)D. 1,25-(OH)_2_-D stimulates bone resorption and intestinal calcium absorption, leading to an increase in serum calcium concentration. The synthesis and secretion of 1,25-(OH)_2_-D is mainly regulated by changes in serum parathyroid hormone (PTH) concentration, which is regulated by the serum calcium concentration, as well as by serum phosphate concentration and by itself. Fibroblast growth factor 23 (FGF23) is also involved in the regulation of 1,25-(OH)_2_-D (3). 1,25-(OH)_2_-D exerts its main biological effects via an intracellular vitamin D receptor (VDR). The VDR has been found in many cell types. Recent detailed analysis has not confirmed the presence of VDR in cardiac and skeletal muscle, but there is an ongoing debate on this issue, as 1,25-(OH)_2_-D has specific effects on, that is, muscle cells (4, 5). The 1,25-(OH)_2_-D–VDR-complex acts as transcription factor in the target cells. The classical targets are the intestinal mucosa cells and the skeleton. In the intestine 1,25-(OH)_2_-D induces the calcium-binding protein (calbindin) and the calcium channel TRPV6 (6). In bone tissue, the role of 1,25-(OH)_2_-D is complex but it is a strong regulator of receptor activator of NF-κB ligand( RANKL) a key molecule in osteoclastogenesis (7). Recently, it has been shown that 1,25-(OH)_2_-D can be produced from circulating 25(OH)D locally in other cells than kidney cells, for example, in osteoblasts. In this way 1,25-(OH)_2_-D can exert its effects in an autocrine or paracrine manner (for review, see the study of Norman and Bouillon [8]).

**Fig. 1:**
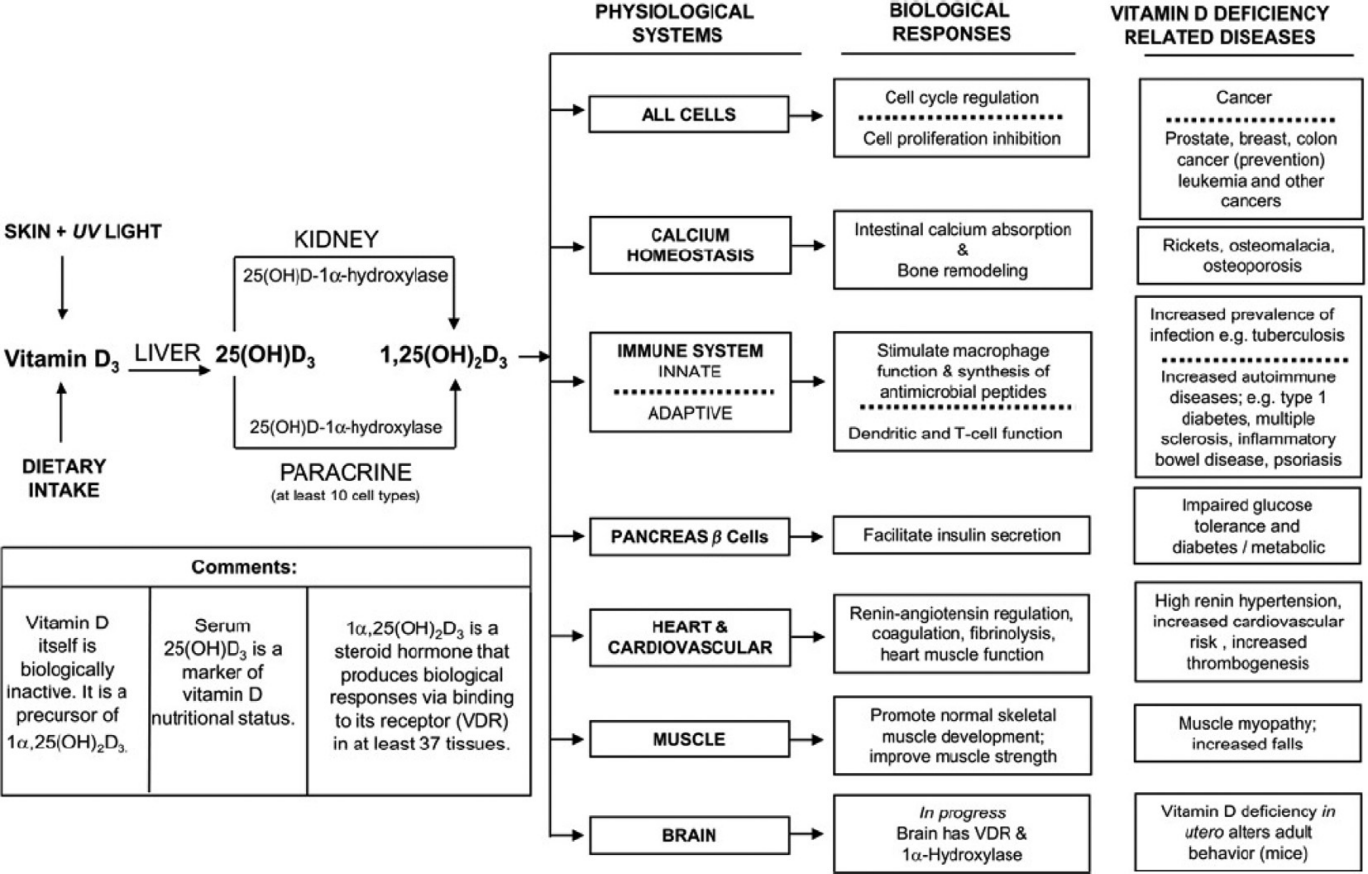
Overview of vitamin D and its role on physiological systems and the biological responses as well as possible vitamin D-related diseases. The three columns on the right side, respectively, indicate the following: physiological systems (the six physiological systems that the essential nutrient vitamin D_3_ supports by its metabolism to 25(OH)D_3_ and 1α,25(OH)_2_D_3_); biological responses (examples of biological responses generated by 1α,25(OH)_2_D_3_ in the six physiological systems); and vitamin D-deficient-related diseases (identifies for each system some of the disease states that are associated with an inadequate vitamin D nutritional status) (8).

1,25-(OH)_2_-D has important roles in many physiological systems beside calcium homeostasis: the immune system, the pancreatic beta-cells to name a few and has distinct biological responses in the related cells. Fig. 1 displays some of the roles of 1,25-(OH)_2_-D in physiological systems and the biological responses as well as diseases and health outcomes that could be related to vitamin D deficiency (8).

### Vitamin D status and vitamin D sources in the Nordic countries

#### Denmark

The vitamin D status in Denmark has been evaluated in a few studies. Andersen et al. (9) performed a cross-sectional study in five different European countries, one of which was Denmark. They showed that 51% of teenage girls had 25(OH)D concentrations lower than 25 nmol/l and 93% had concentrations lower than 50 nmol/l in the winter. In addition, 17% of elderly women had concentrations lower than 25 nmol/l and 55% concentrations lower than 50 nmol/l. The median vitamin D intake from diet was 2.4 and 3.4 µg in the girls and women, respectively. Vitamin D supplements were used by 34% of the girls and 62% of the elderly women. In a recent study, Thuesen et al. (10) evaluated the vitamin D status in 6,146 randomly selected individuals aged 30–60 years that participated in a health examination in 1999–2001. The overall prevalence of vitamin D deficiency (25(OH)D <25 nmol/l) and insufficiency (25(OH)D < 50 nmol/l) was 13.8 and 52.2%, respectively. A marked seasonal variation was seen in the 25(OH)D concentrations, the median 25(OH)D concentrations were lowest in February and highest in August. Estimated dietary intake of vitamin D ranged between 0.2 and 22.5 µg/day (median 3.0 µg/day, *n*=6,224). Data on the use of supplements were not collected and the vitamin D sources were not explored in this study. Serum 25(OH)D concentrations were not associated with the estimated dietary intake of vitamin D. The Pakistanis are a large immigrant population in Denmark. In a study in girls, women and men of Pakistani origin, Andersen et al. (11) showed in a cross-sectional study spanning over January–November that the incidence of severe vitamin D deficiency was very common. Eighty-one percent of the girls and 84% of the women had 25(OH)D concentrations below 25 nmol/l and 46% of the girls and 40% of the women were below 10 nmol/l. Sixty-five percent of the men had 25(OH)D concentrations below 25 nmol/l and 13% were below 10 nmol/l. Almost all persons had 25(OH)D concentrations below 50 nmol/l. Use of vitamin-D-containing supplements had a positive association with S-25(OH)D for both men and women. Dietary vitamin D intake was 2.2 µg/day in men and 1.7 µg/day in women. Based on these reports, the vitamin D status in Denmark seems to be problem both in the native Danish population but especially in the Pakistanis.

#### Iceland

In Iceland, cod liver oil is an important and traditional source of vitamin D, especially for children and the older generation, presently supplying 48% of total vitamin D from foods according to the National Nutrition Survey. Fatty fish and fortified fats are also important sources. Vitamin D intake varies considerably within the population, with 10% of adults having a habitual intake of ≤3.1 µg/day, while 10% have a habitual intake of ≥21.6 µg/day of vitamin D (12). Supplement use contributes greatly to this variation. Young adults, aged 18–30 years and not taking supplements, have a mean intake of 3.9 µg/day of vitamin D, while the same age group taking cod liver oil has a mean intake of 13.5 µg/day (12). The significance of supplement use, including cod liver oil, is also reflected in vitamin D status in Iceland, with serum 25 OHD concentrations averaging <28 nmol/l in February–March in adult men and women not taking supplements, compared with 48 nmol/l for those taking cod liver oil or other vitamin-D-containing supplements (13). The authors conclude that supplements are needed for adequate vitamin D status during winter in northern regions. Icelandic food and nutrition recommendations from 2004 advise the use of vitamin D supplements or cod liver oil (14) and pre-schools commonly supply cod liver oil to children throughout the year.

#### Finland

The vitamin D intake and vitamin D status has been low in Finland in all age groups. The authorities have, however, introduced fortifications schemes to broaden the sources of vitamin D in the population. In 2003, the Ministry for Trade and Affairs, based on simulations, recommended that all fluid milk products should be fortified with 0.5-µg vitamin D_3_/100 g, and all spreads with 10 µg/100 g (previously 7.5 µg/100 g). The effect of this fortification has been evaluated in a large population study of about 650 participants (aged 4–74 years) with blood samples and other data from 2002 and 2004. The median daily intake increased, for example, by 1.8 µg in 27–66 year olds and the increase in the 25(OH)D concentration was 7.0 nmol/l (15). In those using fluid milk products, the impact on intake and vitamin D status was considerable. The main sources were fish/fish products and fortified milk products, the importance of which is dependent on the age groups. The use of supplements was important as a source of vitamin D in all age groups (15). There were some groups that were still at risk – small children, pubescent girls, and young and middle-aged women. In the Findiet 2007 study, the mean daily dietary vitamin D intake of women aged 25–65 years was 5.2 µg and in 65 to 74-year-old women 6.5 µg, whereas it was 7.1 and 9.0 µg in the corresponding age groups for men (16).

In 2010, the National Board of Nutrition increased the recommendation for fortification to 1 µg/100 g fluid milk products and for spreads 20 µg/100 g. Moreover, the authorities (National Board of Nutrition; Institute of Welfare and Health; Finnish Paediatric Society) recommend since 2011 that children and youths aged 3–18 years should take a daily 7.5 µg vitamin D supplement all around the year, whereas children younger than 3 years should take a 10-µg daily supplement. Noteworthy is that vitamin D supplements have been recommended to children younger than 3 years for decades in Finland, but it has largely been given only to children during their first year of life (17) Currently, there are no published studies in Finland from 2010 to show what the actual intake and vitamin D status is in the Finnish population. Regarding ethnic groups, a recent study by Islam et al. (18) has shown that Bangladeshi women but especially Somali women has a very low vitamin D status in Finland.

#### Norway

The main dietary sources of vitamin D in the Norwegian population are fatty fish, fortified margarine and butter and cod liver oil supplements (19). In addition, it is common to take other vitamin D supplements. The use of cod liver oil supplements represents a long dietary tradition in Norway. A nationwide dietary survey found that 45% of middle-aged women reported cod liver oil supplement use (20). However, the use of this supplement has been found to be less among the younger population. The contribution of cod liver oil supplement to increase vitamin D intakes in Scandinavia compared to southern Europe has been described (21). A systematic review has been conducted by Holvik et al. (22) for available literature on vitamin D status in Norway. They concluded that the vitamin D status was sufficient for the majority in the general population (25(OH)D ≥50 nmol/l was considered as optimal) and that available data suggest that the vitamin D status in Norway is better than more southern locations in Europe. In spite of this, some have insufficient 25(OH)D concentrations, and that vitamin D status dropped in late winter, also in southern Norway. Some vulnerable groups were identified, that is, non-western immigrants and the elderly, especially those living in nursing homes. A working group on vitamin D in the Norwegian population, nominated by the National Council of Nutrition, recommended in their report (19) an increased fortification of foods, in particular milk, in order to improve the vitamin D status in the population including vulnerable groups.

#### Sweden

The vitamin D intake of the adult Swedish population was reported in 1998 in the national survey, Riksmaten (23). The median daily vitamin D intake spanned from 4.0 µg/day in 17 to 24-year-old women to 5.6 µg in women aged 65 years and older. Correspondingly, the median daily vitamin D intake in 17 to 24–year-old men was 4.9 µg and 7.0 µg in men older than 65 years. The main sources were dietary fat, fish and fish products and fortified milk products. Serum 25(OH)D concentrations were not measured. A similar survey was performed in 2010–2011, but the results are not available. Vitamin D intake and status has been studied in children. In ‘Riksmaten–barn 2003’ (24), a nutrition survey in children, found that the mean intake was 6.6, 5.0 and 4.6 µg in 4-year-olds, 2nd grade and 5th grade, respectively. The higher intake in the youngest was due to the fact that 21% of them got vitamin D supplements and 28% ate fortified porridge. In a recent study, Eriksson and Strandvik (25) found that the mean 25(OH)D concentration was 76 and 68 nmol/l in 4- and 8-year olds, which could be considered satisfactory. However, a larger percentage (ca. 30%) of the older children had concentrations less than 50 nmol/l than the younger ones (<10%) and 65% of the older boys and 55% of the older girls had concentrations <75 nmol/l whereas the numbers were 50 and 40%, respectively in the younger age groups. The authors state that the comparably high 25(OH)D concentrations are due to the fact the children up to the age of five regularly get vitamin D supplementation.

### Research/key questions for vitamin D

The selection of outcomes was based on our knowledge of the vitamin-D-related scientific literature. The NNR5 Working Group commented on and approved of the research questions.

The research questions for this systematic review were as follows:

1. What is the effect of vitamin D from different sources on serum 25(OH)D concentrations?
2. What is the relationship between 25(OH)D concentrations and different outcomes in different populations and age groups?
3. What is the effect of dietary vitamin D intake on different outcomes in different populations and age groups?
4. What is the effect of supplemental vitamin D on different outcomes in different populations and age groups?
5. What is the effect of sun or UVB exposure on different outcomes in different populations and age groups?
6. What is the UL (tolerable upper intake level) for vitamin D for different health outcomes in different populations and age groups?
7. What are the interactions of vitamin D with calcium intake on different health outcomes in different populations and age groups?
8. Which is the interaction of vitamin D intake or vitamin D status with vitamin A intake or vitamin A status on health outcomes in different populations and age groups?

## Methods

### Definitions

The exposures were:

*For research question 1:* diet/dose; sun exposure/season; supplements/dose/intervals; obesity; pregnancy/lactation.

*For research questions 2–8:* dietary vitamin D, fortified foods, supplementation and sunlight (natural UV irradiation) exposure, serum 25-hydroxy-vitamin D concentration, vitamin A intake.

Serum or plasma 25(OH)D-concentration was used as an *indictor of exposure* in research questions 2–8.

The following outcome measures were included:

*For research question 1*: 25(OH)D. *For research questions 2–5, 7 and 8*: Pregnancy outcomes and growth, bone health (all fractures, hip fractures, vertebral fractures, bone mineral density/osteoporosis, bone mass, bone quality, rickets, osteomalacia, dental health); muscle strength; falls; all cancers, breast cancer; colorectal cancer; prostate cancer; diabetes type I; diabetes type II; multiple sclerosis; obesity; total mortality; hypertension/blood pressure; cardiovascular disease (CVD) clinical outcomes; infections.

*Research question 6:* calcium metabolism: hypercalciuria, hypercalcemia; soft tissue calcification; renal outcomes vascular outcomes; mortality; adverse events reported in RCTs

Th*e following life stages* were included: infants, children, adolescents, adults, postmenopausal women, elderly, the very old.

### Search methods and terms

Two expert reference librarians designed and conducted the electronic search strategy based on the research questions provided by the four investigators. The following electronic databases were searched: MEDLINE and Swemed. The search was conducted using medical subject heading terms (MESH) (see Appendix 1). The search was done in two batches, the first covering January 2000–March 2010 and the second March 2009–February 2011. In the first search, the investigators focused only on SLRs) and in the second on systematic reviews and randomized control trials (RCTs) published after March 2009. Furthermore, we used snowballing for SLRs and RCTs published after that and until May 2012.

### Selection of articles/studies

The investigators screened all abstracts from both searches in pairs, and after that all four investigators made a common decision on the full-text articles to be acquired from the librarian. From the batches of full-text articles, we included those who met the criteria for SLRs. As regards RCT studies, only studies from Europe and North America were included. The full-text articles were examined in pairs and the four investigators made a common decision on which articles should be included and which to exclude. Eligible criteria for full-text articles were SLR, matching the research questions and healthy populations, not patients or medication, and not meta-analyses.

### Quality assessment of studies

Results of *systematic reviews and meta-analysis* were quality assessed and evaluated using the NNR5-modified AMSTAR quality assessment tool and incorporated in the evidence tables. Quality assessment of the RCTs was made according to the NNR guidelines (2). The quality assessment methods of the studies included in the SLRs differed. The Jadad scale is one of the instruments used to assess the quality of RCTs and is referred to in some of the SLRs in this review (26).

### Reporting of evidence

The evidence is reported in the evidence tables (Appendix 2) and the summary tables (Appendix 3).

## Results

### Result of search

**Fig. 2:**
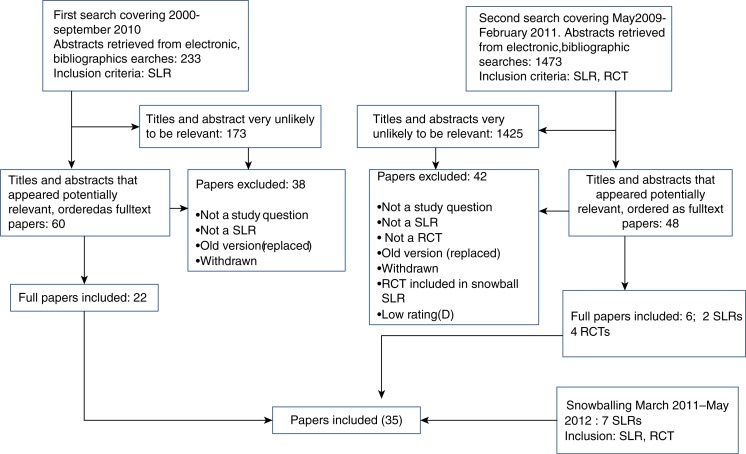
Flowchart of study selection.

*In total 1706 abstracts were screened* (Fig. 2). The search was done in two batches, the first covering January 2000–September 2010 and the second covering May 2009 to February 2011 In the first search, the investigators focused only on SLRs and in the second on SLRs and RCTs. Furthermore, the authors used snowballing for SLRs and RCTs published between March 2011 and May 2012. We primarily identified 108 studies for further consideration, whereas 1,598 studies were excluded. Finally, we included 28 studies based on the literature search and 7 by snowballing, 35 in total. The included studies are listed in the reference list and the excluded studies are listed in Appendix 4. The characteristics and quality of the SLRs and included RCTs are presented in Appendix 2, respectively. The results of the studies are presented in specific summary tables 1–23 (Appendix 3).

Noteworthy is, that two extensive SLRs, Cranney et al. (27), focusing on the effectiveness and safety of vitamin D, calcium in relation to bone health and Chung et al. (28) focusing on vitamin D, calcium and health outcomes, were performed for the North American vitamin D and calcium recommendations (29). Chung et al. (28) included material from Cranney et al. (27), and in some cases built their conclusions on this earlier evidence report.

### The effect of vitamin D from different sources on serum 25-OHD concentrations (Research question 1)

#### Effect of dietary vitamin D on 25-hydroxy-vitamin D concentration

We did not identify any SLR on the relationship on dietary vitamin D from natural sources and 25(OH)D-concentration.

#### Effect of fortified foods on 25-hydroxy-vitamin D concentration

We identified two SLRs on the effect of fortification (28, 30). For more information, see summary table 1. Moreover, O'Donnell et al. (31) published a paper based on part of the same material as Cranney et al. (27), which is included in this analysis. Chung et al. (28) did not perform a new SLR but based their conclusions on Cranney et al. (27).

Cranney et al. (27) included 13 RCTs that studied the effect of fortified dietary sources of vitamin D on circulating 25(OH)D-concentrations. Two of the 13 trials did not provide the vitamin D content of the dietary source and were excluded, thus 11 studies were included in the analyses. They studied a total of 1,281 subjects (697 interventions, 584 controls). All trials were carried out in adults. The quality of 6 out of 11 trials was scored ≥3 on the Jadad scale (26).

The vitamin D dietary interventions included fortified milk, nutrient dense fruit and dairy-based products, high vitamin D diet, fortified orange juice, fortified cheese and fortified bread. The only RCT with a factorial design had two other intervention groups that included an exercise program and a combined program of exercise and nutrient dense products. The type of vitamin D administered was vitamin D_3_ in eight trials and was not specified in three. The vitamin D intake was 5–25 µg/day. Seven trials also specified the calcium content within the dietary intervention. The duration of the intervention ranged from 3 weeks to 24 months. Compliance was reported in four trials and was stated to be >85%.

Meta-analysis was conducted to quantify the effects of dietary sources with vitamin D with/without calcium versus placebo or calcium on serum 25(OH)D concentrations. Seven of the 11 included trials that reported (or provided sufficient data to calculate) the absolute change in total 25(OH)D or 25(OH)D_3_ concentrations were included in the meta-analysis.

Combining all seven trials that investigated the effect of food fortification or dietary sources of vitamin D with or without calcium versus control was not possible due to heterogeneity of the treatment effect. However, the individual weighted mean differences demonstrated a clear trend toward a significantly higher absolute change in serum 25(OH)D concentration in the treatment group versus the control.

The positive direction of the treatment effect of dietary interventions with foods fortified with vitamin D was consistent. Those trials with low baseline 25(OH)D concentrations (i.e. <50 nmol/l) demonstrated a greater percent increase in 25(OH)D concentrations at the end of study compared to trials with higher baseline 25(OH)D concentrations (i.e. >50 nmol/l). The authors stated that observations from such indirect comparisons need to be interpreted cautiously due to differences in baseline characteristics of the study populations, the bioavailability of the vitamin D in the various food sources and the different measures of serum 25(OH)D used.

Cranney et al. (27) concluded that

Eleven of the thirteen identified trials on food fortification and circulating 25(OH)D provided the vitamin D content (5–25 µg) of the dietary source. Most trials used dairy products as the source of fortified foods. Food fortification with vitamin D resulted in significant increases in serum 25(OH)D concentrations with the treatment effect ranging from 15 to 40 nmol/L. The combined effect of fortified food from two trials with vitamin D_3_ doses equivalent to 10–12 µg/d was 16 nmol/L (95% CI 12.9, 18.5). It was not possible from these trials to determine if the effect of food fortified with vitamin D on serum 25(OH)D concentrations varied by age, BMI or ethnicity.

Black et al. (30) performed an SLR based on 16 RCTs from 15 publications of which 8 were included in the Cranney et al. report (27). Five studies scored <3 on the Jadad scale and the rest scored ≥3(26). Compliance rate was reported in 10 studies, which is important in food-based studies, but not included in the Jadad scale. The heterogeneity among the studies was high. The authors did not distinguish between vitamin D_3_ and vitamin D_2_ in the analyses. The authors concluded that

foods fortified with vitamin D increased circulating 25(OH)D concentrations in a dose-dependent manner. In addition they concluded that the treatment effect was higher in studies using doses ≥10 µg/d, in studies performed at latitudes 40 degrees and where baseline 25(OH)D concentrations were less than 50 nmol/l. Moreover, the authors calculated that a mean individual daily intake of about 11 µg vitamin D from fortified foods increased serum 25(OH)D concentrations by 19.4 nmol/l on an average corresponding to an average 1.2 nmol/l increase for each 1 µg vitamin D ingested.

#### Effect of supplementation on 25-hydroxy-vitamin D concentration

We identified two SLRs (27, 32), for details see summary table 2. Chung et al. (28) did not perform a new SLR but based their conclusions on Cranney et al. (27). They included further analyses of dose response.

Cranney et al. (27) analyzed the effect of vitamin D supplementation on circulating 25(OH)D concentrations in different age groups, and the the result are shown below.

##### Infants

Seven trials included term infants. Four trials used vitamin D_2_, vitamin D_3_ was used in one and in three trials no information was given on the form of vitamin D. Most trials were of lower methodological quality. The authors concluded that

one trial suggested that 5 µg of vitamin D_2_ may not be enough to prevent vitamin D deficiency, in some infants residing at northern latitudes. A dose-response was noted in this same trial (2.5, 5, 10 µg/day). Consistent responses to vitamin D supplementation were noted across the seven trials, and some trials suggested that infants, who are vitamin D deficient, may respond differently and require higher doses of vitamin D.

##### Pregnant women and lactating mothers

Six small trials of vitamin D supplementation in pregnant or lactating women were included. Three trials used vitamin D_2_ and three used vitamin D_3_. All trials were of low methodological quality. The authors concluded that

25–90 µg/d of vitamin D_2_ and 25 µg/d of vitamin D_3_ resulted in significant increases in serum 25(OH)D concentrations in lactating mothers and in cord blood. One trial found that supplementation of lactating mothers with 25 µg of vitamin D_2_ during winter months did not increase serum 25(OH)D concentrations in the infants.

##### Children and adolescent populations

The authors found four trials that examined the effect of vitamin D on 25(OH)D in children or adolescents with doses ranging from 5 to 50 µg of vitamin D_3_/day in three trials or 10 µg of vitamin D_2_ in one trial. The study quality was rated ≥3 in three trials on the Jadad scale (26). The authors concluded that

there were consistent increases in 25(OH)D concentrations ranging from 8 nmol/L (with 5 µg of vitamin D_3_), 16.5 (with 15 µg) to 60 nmol/L (50 µg).

##### Premenopausal women and younger men

Ten small trials included premenopausal women and younger males. Three trials compared vitamin D_2_ to vitamin D_3_ in healthy young adults. Doses of vitamin D_3_ ranged from 15 to 250 µg/day and for vitamin D_2_ the doses were 100 µg/day or 1,250–2,500 µg for one dose. The methodological quality of eight of the 10 trials was poor. The authors concluded that

Three trials found that vitamin D_2_ and D_3_ in healthy adults may have different effects on serum 25(OH)D concentrations. Vitamin D_2_ appeared to have a smaller effect on serum 25(OH)D, which may have been due to more rapid clearance and/or different metabolism than vitamin D_3_. One trial compared 2500 µg vitamin D_2_ orally versus injection and found a greater variability in response with the intramuscular preparation. A dose-response effect was noted in those trials that used multiple doses of vitamin D_3_.

##### Postmenopausal women, older men, and elderly populations

Forty-four trials were conducted exclusively in postmenopausal women and older men. Fourteen of these were performed in elderly populations living in long-term care or nursing homes. One trial was in early postmenopausal women. Doses ranged from 2.5 to 1,000 µg/day of vitamin D_3_ and 225 µg vitamin D_2_/day. In three studies, single doses of 2,500–7,500 µg as injections were used. One trial was conducted in African American women. The methodological quality was ≥3 in 24 trials. One trial found that wintertime declines in 25(OH)D concentration were prevented with 12.5 µg of vitamin D_3_ daily. A dose response with increasing doses of vitamin D_3_ was noted. The authors also performed a meta-analysis of 16 of the 44 trials in postmenopausal women, older men, and elderly populations that investigated the effect of oral vitamin D supplementation with or without calcium versus no treatment, placebo or calcium on serum 25(OH)D concentrations. They concluded that

treatment effect of oral vitamin D_3_ supplementation increases with increasing doses. Meta-regression results demonstrated a significant association between dose and serum 25(OH)D levels. The meta-regression results suggested that 2.5 µg/d of vitamin D_3_ will increase the serum 25(OH)D concentrations by 1–2 nmol/L. This suggests that doses of 10–20 µg daily may be inadequate to prevent vitamin D deficiency in at-risk individuals. Vitamin D_3_ doses of 17.5 µg daily or more significantly and consistently decreased serum concentrations of PTH in vitamin D deficient populations. Given the limitations in the measurement of 25(OH)D concentrations and the lack of standardization and calibration, it is difficult to suggest precise recommendations for adequate intakes, especially since optimal levels of serum 25(OH)D have not been defined.

Chung et al. (28) further analyzed the effect of vitamin D supplementation on changes in serum 25-OHD concentration based on the results from Cranney et al. (27). They plotted the net changes in serum 25(OH)D concentration against the doses of vitamin D supplementation using data from 26 RCTs with 28 comparisons in adults. Only RCTs of daily vitamin D_3_ supplementation (doses ranged from 5 to 125 µg/day) alone or in combination with calcium supplementation (doses ranged from 500 to 1,550 mg/day) that provided sufficient data for the calculations were included in the plot. The studies had varied compliance rates in the vitamin D intake; limited or no adjustment for skin pigmentations, calcium intake, or background sun exposure; different vitamin D assay methodologies and measurement variability. They stated that these factors increased the heterogeneity and limited the usefulness of an overall summary estimate for an intake dose response in serum 25(OH)D concentration. Chung et al. (28) concluded that

a relationship between increasing doses of vitamin D_3_ with increasing net change in 25(OH)D concentration was evident in both adults and children, that the dose-response relationships differed depending on study participants’ serum 25(OH)D status (≤40 vs. >40 nmol/L) at baseline, and depending on duration of supplementation (≤3 vs. >3 months). Vitamin D_2_ supplementation was more commonly used in RCTs of infants and pregnant or lactating women, than vitamin D_3_ supplementation. Results showed that supplementation of vitamin D_2_ significantly increased 25(OH)D concentrations in infants, lactating mothers and in cord blood.

Cashman et al. (32) included 44 RCTs in their systematic review. In the analyses, priority was given to data from winter-based RCT (*n*=12) performed at latitudes higher than 49.5 degrees *N*. Six of the 12 RCTs were included in Cranney et al. (27) and had a Jadad score ≥3 (26), the rest were not quality assessed but were included in the final IoM report (29). The authors concluded that

A combined weighted linear model meta-regression analyses of natural log total vitamin D intake (diet and supplemental vitamin D) versus achieved serum 25(OH)D-concentration in winter produced a curvilinear relationship. Use of non-transformed total vitamin D intake data (maximum 35 µg/d) provided for a more linear relationship. Although inputting an intake of 15 µg/d (i.e. the US RDA) into the 95% lower CI curvilinear and linear models predicted a serum 25(OH)D of 54.4 and 55.2 nmol/l, respectively, the total average vitamin D intake that would achieve 50 (and 40) nmol/l serum 25(OH)D was 8.9 µg (2.8) and 12 (6.5) µg/d, respectively. Inclusion of 95% range in the model to account for inter-individual variability increased the predicted intake of vitamin D needed to maintain serum 25(OH)D ≥50 nmol/l to 23.25 µg/d.

The authors also stated that

these results should be interpreted with caution because of the few data points in the analysis.

### Vitamin D and different health outcomes (Research questions 2–4)

#### Vitamin D and pregnancy

We found two SLRs on pregnancy-related outcomes and vitamin D that met our inclusion criteria (28, 33). The reviews are presented in summary table 3.

Chung et al. (28) evaluated one nested case-control study of healthy, nulliparous pregnant women (*n*=274) that were followed from less than 16 weeks of pregnancy to delivery. Women who subsequently developed preeclampsia had lower adjusted mean 25(OH)D concentrations than controls. Early pregnancy maternal 25(OH)D concentrations below 37.5 nmol/l were associated with a fivefold increased risk of preeclampsia. Furthermore, babies of preeclamptic mothers were twice as likely to have serum concentrations below 37.5 nmol/l compared with controls. None of these associations varied with race or ethnicity. The study was rated B.

De-Regil et al. (33) reviewed six randomized trials including 1,023 pregnant women, in a report that updates a previous Cochrane report on vitamin D supplementation and maternal and neonatal outcomes. Intended maternal outcome measures were preeclampsia, gestational diabetes and vitamin D status at term. Infant outcome measures were preterm birth and low birth weight. In addition, there were a series of secondary intended outcome measures, including cesarian sections, maternal hypertension and Apgar score. Most of the studies were done in the 1980s while one was from 2008 and the dose of vitamin D given on a daily basis ranged from 20 to 30 µg. Three trials also included high doses in one of their arms: two of them used a single dose of 5,000 µg in the third trimester and one gave 15,000 µg twice during pregnancy. Five of the studies, including 623 women supplied vitamin D alone while one study of 400 women gave vitamin D in combination with calcium. None of the included studies reported on gestational diabetes or preterm birth. Preeclampsia was only reported in the one study giving both calcium and vitamin D, and found no difference in risk between the women receiving supplements compared with the placebo group.

The authors’ conclusions were as follows:

The use of vitamin D supplementation during pregnancy improves vitamin D concentrations as measured by 25-hydroxyvitamin D at term. However, the clinical significance of this finding is yet to be determined as there is currently insufficient high quality evidence relating to the clinical effects of vitamin D supplementation during pregnancy. Good quality studies are needed to determine the usefulness and feasibility of this intervention as a part of routine antenatal care.

#### Vitamin D and growth

One SLR (28) was identified evaluating seven interventions and two observational studies on vitamin D and growth in newborns, infants, or children. The review is presented in summary table 4. Two interventions included in the review, where pregnant women in India received 15,000 µg in the 7th and 8th months of pregnancy, were the only intervention trials reporting statistically significant effects of vitamin D supplements on growth. The studies were rated C as important aspects of the methodology were not reported. Dietary vitamin D intakes of these mothers were estimated to be less than 0.7–0.9 µg/day. A British trial of 126 Asian women receiving 25 µg/day during the third trimester reported no effects on birth weight or length even though there was an insignificant reduction in the number of low birth-weight infants in the intervention group. Similarly, no significant effect was demonstrated by French, Chinese, or Australian trials. All of these trials were rated C for methodological quality except for the British trial which was rated B. Two cohort studies were also evaluated, one British and one Australian. Neither study showed significant associations between maternal serum 25(OH)D and growth of the offspring. The authors are *cautious in their conclusions regarding the evidence on vitamin D related to growth, citing lack of methodologically solid studies*. Chung et al. (28) also reviewed the relationship between vitamin D and calcium and growth. They found one C-rated study from India comparing vitamin D and calcium supplementation in women in their third trimester to no supplementation. Infants of the women receiving supplementation were significantly heavier.

#### Vitamin D and bone health

##### Rickets

We identified two SLRs that met our inclusion criteria (28, 34). For the results of the studies see summary table 5.

Chung et al. (28) built on and updated the AHRQ-Ottawa evidence-based report of Cranney et al. (27), reviewing concentrations of 25(OH)D related to established vitamin-D-dependent rickets in infants and young children. As an updated search did not identify any new studies, they simply referred to Cranney et al. (27). In 6 of the 13 studies reviewed, mean or median 25(OH)D-concentration in children with rickets was <27.5 nmol/l, whereas it was between 30 and 50 nmol/l in the other studies. Most studies were conducted in developing countries with low calcium intake. Low calcium intake can influence the relationship between 25(OH)D and rickets, and the 25(OH)D threshold for rickets in populations with high calcium intake (e.g. North America) is unclear. The Cranney et al. (27) report thus concluded:

There is fair evidence for an association between low serum 25(OH)D and established rickets, regardless of assay type (RIA, CPBA, HPLC). There is inconsistent evidence to determine if there is a threshold concentration of serum 25(OH)D above which rickets does not occur.

In a Cochrane review by Lerch and Meissner (34), the aim was to evaluate the effects of interventions on the prevention of nutritional rickets in term-born children. The review was limited to studies performed in the last 50 years. Only four trials were included and in two of them no rickets occurred. The reference was placebo or no intervention. In a Turkish trial, vitamin D showed a reduced risk of rickets compared to no intervention. In a Chinese trial, a combined intervention of vitamin D and calcium supplementation and nutritional counseling reduced the risk of rickets compared to no intervention. They conclude:

There are only few studies on the prevention of nutritional rickets in term born children. Until new data become available, it appears sound to offer preventive measures (vitamin D or calcium) to groups of high risk, like infants and toddlers; children living in Africa, Asia or the Middle East or migrated children from these regions into areas where rickets is not frequent.

##### Fractures

We identified three systematic reviews that met our inclusion criteria (27, 35, 36). For the results of the studies see summary table 6.

The Cochrane review by Avenell et al. (35), comprising postmenopausal women and men over 65 years of age, concluded that based on available RCTs, it appears unlikely that vitamin D alone is effective in preventing hip fracture, vertebral fracture, or any new fracture. However, a significant reduction in the incidence of hip fracture in those receiving vitamin D (dose 10–20 µg/day) and calcium versus placebo or no treatment was found. Subgroup analysis showed a significant reduction in the subgroup of institutional residents but not in community dwellers. However, the difference between the two subgroups was not statistically significant. The reduction in incidence of non-vertebral fractures was not significant in those given vitamin D and calcium. However, in the subgroup analysis on residential status, a statistical significant effect was found in the institutional residents’ subgroup but not in community dwellers. There was no reported effect of vitamin D and calcium on clinical vertebral fracture. Avenell et al. (35) reported on the scientific quality on nine different items with scores from 0 to 2. No overall score was given.

The SRL by Vestergaard et al. (36) mainly refers to Avenell et al. (35) described above. In addition, the results from the DIPART study (37) are referred to. In this patient-based pooled analysis of seven major vitamin D fracture trials with 68,500 participants, no significant effect of vitamin D alone compared to placebo/no vitamin D was found for any fracture or hip fracture (doses of vitamin D 10–20 µg/day). However, the overall risk of fracture was reduced in those given combined supplementation with vitamin D and calcium compared to placebo/no vitamin D. The risk of hip fracture was hazard ratio (HR) 0.84, 95% CI 0.70–1.01, later corrected to HR 0.83, 95% CI 0.69–0.99 due to a coding error in the original publication, conf. BMJ 2010; 340:b5463). One of the studies included in DIPART included a drug review in those receiving vitamin D and calcium. Additional analysis excluding this study from the pooled analysis attenuated markedly the effect of vitamin D and calcium on hip fractures but not on all fractures. Vestergaard et al. (36) also reported on the results from an RCT published in 2010 (38) in 2,258 women, aged 70 years or older. A single high dose of vitamin D_3_ (12,500 µg) µg or placebo was given orally once a year over a period of 3–5 years. Vitamin D_3_ significantly increased the risk for any fracture compared with placebo. In addition, the incidence of falls was significantly increased in the vitamin D_3_ group compared to placebo. The increased incidence of falls was most prominent in the first 3 months after dosing with vitamin D_3_. Vestergaard et al. (36) concluded that ‘concerning fracture prevention in postmenopausal women, vitamin D plus calcium is likely to be beneficial, whereas vitamin D alone is unlikely to be that’.

The SLR by Chung et al. (28) included and updated the Cranney et al. (27) evidence-based report and most of the results are presented/specified in the Cranney report. When we refer to the Chung et al. (28), this also includes the Cranney et al. report (27).

It was concluded that based on observational studies, the evidence was inconsistent for an association between serum 25(OH)D and the risk of fractures. Combining the results from 13 RCTs intervening with vitamin D_2_ or D_3_ (with or without additional calcium supplementation), a non-significant reduction in total fractures was found. Studies intervening with vitamin D alone showed no effect on fracture incidence by meta-analyses. However, meta-analyses of studies intervening with vitamin D_3_ (10–20 µg/day) plus calcium, showed a reduction in the risk of total fractures and hip fractures. In a subgroup analysis, a significant effect was only present in institutionalized elderly. It was stated that one possible explanation for this was that the studies in institutionalized elderly achieved on average a higher 25(OH)D concentration at the end of the study than the studies in community dwellers. The combined result for studies with higher S-25(OH)D at follow-up (≥74 nmol/l) was a significant reduction in total fractures, which was not the case for studies achieving <74 nmol/l. Cranney et al. (27) stated that this should be interpreted with caution as 25(OH)D was only determined in subsamples and there was variability in measurement methods.

None of the trials in the meta-analysis were performed in premenopausal women.

Cranney et al. (27) concluded *that*

Vitamin D_3_ combined with calcium is effective in reducing fractures in institutionalized populations, whereas the evidence for community dwellers is less strong.

The Cranney et al. (27) report was updated in the Chung et al. (28) report by a new literature search, and a new RCT reporting on fracture showing no effect of an intervention with vitamin D_2_ alone versus placebo was identified. They also identified an RCT quality rated B, performed in women aged 17–35 years, reporting that 20 µg vitamin D/day combined with a daily supplementation of 2-g calcium compared to placebo reduced the risk of stress fracture from military training.

##### Bone mineral density and bone mineral concentration

We identified two systematic reviews that met our inclusion criteria (28, 39). For the results of the studies, see summary table 7. In addition, we identified one RCT in the second search (40).

Chung et al. (28) included the Cranney et al. (27) evidence-based report, and most of the results are presented and specified in the Cranney et al. (27) report. When new data were identified in the update made by Chung et al. (28), this is mentioned in the text and/or in the summary in table 7.

Cranney et al. (27) addressed whether specific concentrations of S-25(OH)D were associated with bone health outcomes in infants, older children and adolescents, pregnant and lactating women, and postmenopausal women and elderly men. They also addressed the evidence regarding the effect of vitamin D supplementation on bone density in women of reproductive age and postmenopausal women and elderly men. Moreover, they also reported on the association between S-25(OH)D and S-PTH. Details are given in the summary tables (summary table 7). They state the following:

##### Infants

There was fair evidence for an inverse relation between S-25(OH)D and S-PTH at low concentrations of 25(OH)D. A threshold may exist around 27 nmol/l. The evidence for an association between specific concentrations of 25(OH)D and bone mineral content (BMC) was inconsistent.

##### Older children and adolescents

No studies assessed the relation between 25(OH)D concentration and fracture. There was fair evidence for an inverse relation between 25(OH)D and s-PTH concentrations. The plateau of PTH concentration ranged from 25(OH)D concentrations of 30–83 nmol/l. They also concluded that there was fair evidence for 25(OH)D concentration being associated with a change in bone mineral density (BMD)/BMC. However, results from two RCTs did not consistently confirm that vitamin D supplementation had an effect. Moreover, they referred to a Finnish RCT (41) in 228 adolescent girls published after they had done their systematic search. The intervention was two doses of vitamin D3 (5 and 10 µg daily) compared to placebo. In per protocol analyses, they reported positive effects on BMC where mean S-25(OH)D >50 nmol/l was achieved in the intervention groups. The results were not statistically significant in the intention to treat analysis. In a cohort study, maternal vitamin D status was weakly related to whole body and spine BMC in children aged 9 years. In a Danish RCT among Pakistani immigrants with very low vitamin D status at baseline, BMD was unaffected by a one-year intervention with 10 or 20 µg/day vitamin D versus placebo.

##### Pregnant and lactating women

During pregnancy, there was fair evidence for a negative association between 25(OH)D and S-PTH concentrations, but insufficient evidence for a relation between 25(OH)D concentration and change in BMD. One good cohort study found no relationship between 25(OH)D concentration and BMD during lactation.

##### Postmenopausal women and older men

In five RCTs and three cohort studies, no association between 25(OH)D concentration and BMD or bone loss was found. A significant association between 25(OH)D concentration and bone loss was found in four cohort studies, most evident at the hip sites. The evidence for a relationship between 25(OH)D concentration and BMD in the lumbar spine was weak. An association between 25(OH)D concentration and BMD was suggested in six case-control studies, and the association was most consistent for femoral neck BMD. They conclude:

There was discordance between the results from RCTs and the majority of observational studies that may be due to the inability of observational studies to control for all relevant confounders. Based on results of the observational studies, there is fair evidence to support an association between serum 25(OH)D and BMD or changes in BMD at the femoral neck. Specific circulating concentrations of 25(OH)D below which bone loss at the hip was increased, ranged from 30–80 nmol/L.

##### Effect of vitamin D supplementation on bone density in women of reproductive age and postmenopausal women and elderly men

Cranney et al. (27) concluded that there was good evidence for vitamin D + calcium supplementation leading to a small increase in spine, femoral neck, total hip, and total body BMD. Based on available studies, it was less certain that vitamin D supplementation alone has an effect on BMD.

In a Cochrane review by Winzenberg et al. (39) including data up to autumn 2009 (6 RCTs, 541 subjects receiving vitamin D, and 343 placebo), the objective was to ‘determine the effectiveness of vitamin D supplementation for improving bone mineral density in children’. The dose administered ranged from 3.3 daily to 350 µg/week. Overall, they did not find any statistically significant effect of vitamin D supplementation on total body BMC, hip BMD, or forearm BMD, whereas a small effect on lumbar BMD was suggested. No statistically significant difference was found between studies using a high or low dose of vitamin D. The difference in effects between studies with high and low baseline S-25(OH)D studies was not statistically significant (total body BMC, *p*=0.09 for difference), although in studies with participants with low S-25(OH)D (≤ 35 nmol/l), a significant effect of supplementation was found for total body BMC and lumbar BMD.

They concluded that

These results do not support vitamin D supplementation to improve bone density in healthy children with normal vitamin D levels, but suggest that supplementation of deficient children may be clinically useful. Further RCTs in deficient children are needed to confirm this.

We also identified one new Danish RCT by Mølgaard et al. (40) with rating B. In this double-blinded RCT, 221 Danish girls aged 10–11 years were randomized to take vitamin D_3_ (5 or 10 µg) or placebo over 1 year. Overall, the intervention had no effect on BMC or BMD (total body and lumbar spine). Compared to the somewhat similar study by Viljakainen et al. (41), which only included girls from September to March (and which found an effect in the compliance controlled analysis), the current study included girls throughout the year.

#### Vitamin D and dental health

We only found one, C-rated, SLR (42) (see summary table 8) including several nutrients with the endpoint being periodontal disease. Only one of the included original papers was on vitamin D. In this cross-sectional study, those in the lowest quartile of 25(OH)D concentration had higher clinical attachment loss compared to those in the highest quintile. The authors conclude *that* ‘the relationship between vitamin D and periodontal disease in elderly is unknown and not well researched’.

#### Vitamin D and falls

We identified seven SLRs (27, 28, 43–47), the results of which are presented in the summary table 9. The definition of ‘falls’ and ‘falling’ varied among the included trials. It should be noted that the trials included in the different SLRs were mainly the same but with some variation due to inclusion and exclusion criteria and timeframes.

Chung et al. (28) included and updated the report by Cranney et al. (27), and most of the results are presented and specified in the Cranney report. This report included two additional RCTs related to vitamin D and falls. Chung et al. (28) concluded that these reports did not change the conclusion made by Cranney et al. (27).

Cranney et al. (27) evaluated the association of 25(OH)D concentrations with falls in postmenopausal women and elderly men. One RCT, three prospective cohorts and one case-control study were included in their analyses. The subjects included in the studies were elderly men and women. The RCT and the cohort studies were of good quality and the case-control of fair quality. The authors concluded that

There is fair evidence of an association between lower serum 25(OH)D concentrations and an increased risk of falls in institutionalized elderly. PTH may be an important confounder. One study suggested a specific serum 25-(OH)D concentration of 39 nmol (l below which fall risk is increased.

Cranney et al. (27) also asked ‘What is the evidence regarding the effect of supplemental vitamin D on falls in postmenopausal women and elderly men?’ A total of 14 trials in 16 publications were included, 12 of which were RCT with a parallel design and 4 using a factorial design. Eleven of the RCTs had a Jadad score ≥3 and the score of the factorial studies was less than three (26). Vitamin D was given by injection in two studies. Oral vitamin D was given as vitamin D_3_ in all but one study. Oral vitamin D was given without calcium in three trials. Meta-analyses were conducted using data from the 12 RCTs. Oral vitamin D did not reduce the risk of falls in comparison to placebo or calcium. Oral vitamin D with calcium showed a reduction in falls as compared to placebo or calcium. Injectable vitamin D_2_ did not reduce the risk of falls in comparison to placebo. The authors summarized that the combined results from 12 trials (*N*=14,101) demonstrated a small reduction in falls with vitamin D_2_/D_3_ (oral or injectable)±calcium. In the two factorial design trials, one demonstrated a significant fall reduction in postmenopausal women taking vitamin D_3_ plus calcium (whereas the other trial did not show a reduction in falls in elderly individuals taking vitamin D_2_). Moreover, the authors summarized that the results from trials examining the effect of supplemental vitamin D on falls are consistent, with 12 of the 14 trials demonstrating a non-significant reduction in falls. However, when combining RCTs (by an intervention method), there is inconsistent evidence regarding the effect of supplemental vitamin D on falls. The combination of 12 trials of either oral or injectable vitamin D_2_/D_3_±calcium did demonstrate a small reduction in fall risk. Combination of eight RCTs of oral vitamin D_2_/D_3_ supplementation with calcium showed a reduction in fall risk, whereas four RCTs of oral vitamin D_3_ alone did not. Subgroup analyses showed a significant reduction in falls upon combining trials of postmenopausal women only. Sensitivity analyses showed a significant reduction in falls when combining: (i) RCTs that explicitly defined falls and the method of fall ascertainment; and (ii) those in which the allocation concealment was unclear. However, combining trials by degree of compliance and loss to follow-up did not result in significant reductions Cranney et al. (27) concluded that ‘there is inconsistent evidence that supplemental vitamin D reduces falls in postmenopausal women and older men’.

Kalyani et al. (43) included 10 RCTs performed in older adults for a systematic review on vitamin D treatment for the prevention of falls. Vitamin D_3_ was used in six studies, vitamin D_2_ in three studies and alfacalcidiol (a synthetic analog) in one study. The methodological quality of the studies was good in general. In pooled analysis, vitamin D (5– 25 µg/day) resulted in 14% fewer falls than calcium or placebo. According to this, SLR the following subgroups had significantly fewer falls: community-dwelling (aged <80), adjunctive calcium supplementation, no history of fractures or falls, duration longer than 6 months, vitamin D_3_, and a dose of 20 µg or greater. Meta-regression demonstrated no linear association between vitamin D dose or duration and treatment effect. Post-hoc analysis including seven additional studies (17 in total) without explicit fall definitions yielded smaller benefit and more heterogeneity but found significant intergroup differences favoring adjunctive calcium over none. The authors concluded that ‘vitamin D treatment effectively reduces the risk of falls in older adults’.

Cameron et al. (44) studied interventions for preventing falls in older people in nursing care facilities and hospitals and included 41 trials (25,422 participants). Five trials tested the effect of vitamin D supplementation on falls. The quality of the studies was generally good. Pooled data from the four studies with 4,512 participants that provided falls rate data show a statistically significant reduction in the rate of falls. Pooled data from all five studies with 5,095 participants did not show a reduction in the risk of falling. The authors stated that caution may be required with interpretation of these pooled data because of statistical and clinical heterogeneity. Two studies investigated vitamin D_3_ and calcium and one vitamin D_2_ in combination with calcium. Two studies compared vitamin D plus calcium to calcium and showed a significant reduction on rate of falls but no reduction in risk of falling. Generally, the baseline serum 25(OH)D concentrations were low in four of these studies. The authors did not distinguish between trials including or not including calcium. *The authors concluded that* 'vitamin D supplementation is effective in reducing the rate of falls in nursing care facilities’.

Gillespie et al. (45) included 13 RCTs focusing on the prevention of falls in older people living in the community. Thirteen studies (23,112 enrolled participants) evaluated the efficacy of vitamin D supplementation, either alone or with calcium co-supplementation for fall prevention. Two studies contained multiple intervention arms. The overall analysis of vitamin D versus control did not show a statistically significant difference in the rate of falls or risk of falling. A subgroup analysis showed no significant difference in either rate of falling or risk of falls in trials recruiting participants with higher falls risk or trials not so doing, and no significant difference in effect size between the subgroups in either analysis. The rate of falls was significantly reduced in trials with participants with lower 25(OH)D concentrations but not in participants not selected. There was a significant difference between these two subgroups with a greater reduction in rate of falls in the subgroup of trials only recruiting participants with lower 25(OH)D concentrations. The authors did not distinguish between trials including or not including calcium. The authors’ conclusion was

Overall, vitamin D does not appear to be an effective intervention for preventing falls in older people living in the community, but there is provisional evidence that it may reduce falls risk in people with low vitamin D levels [25(OHD)].

Michael et al. (46) published an SLR on primary-care-relevant interventions on prevention of falling in older adults. It included nine trial of vitamin D supplementation. Five of these included only women and the proportion of women in the others was 51–80%. Five trials were conducted in populations defined as high risk because of recent falls or vitamin D deficiency. The remaining four studies used populations that were unselected except for ages 65 years or older. All studies were rated as fair quality. The daily oral doses of vitamin D in the intervention ranged from 2.5 to 25 µg/day (median: 20 µg). One study provided a single intramuscular injection of 15,000 µg of vitamin D. Two studies evaluated vitamin D_2_ and the remaining studies evaluated vitamin D_3_. Six trials included calcium supplements with vitamin D. The control groups ranged from no intervention to placebo or calcium supplements only. Vitamin D with or without calcium was associated with a 17% (CI: 11–23%) reduced risk of falling during 6–36 months of follow-up. Trials of vitamin D with calcium compared with no treatment or placebo did not support any added benefit of calcium.

The authors concluded that

There is strong evidence that several types of primary care applicable falls interventions (i.e. comprehensive multifactorial assessment and management, exercise/physical therapy interventions, and vitamin D supplementation) reduce falls among those selected to be at higher risk for falling.

Murad et al. (47) found 26 trials of moderate quality that enrolled 45,782 participants, the majority of which were elderly and female to evaluate the existing evidence on vitamin D use and the risk of falls. Eight studies used vitamin D_2_ and 18 vitamin D_3_ with or without calcium. In 24 studies, vitamin D was given orally and intramuscularly in the remaining two. The results indicated that vitamin D use was associated with a statistically significant reduction in the risk of falls. This effect was more prominent in subjects who were vitamin D deficient at baseline and in studies in which calcium was co-administered with vitamin D. The quality of evidence was low to moderate because of heterogeneity and publication bias, 19 studies were rated high and seven were low. The authors concluded that

vitamin D combined with calcium reduces the risk of falls. The reduction in studies without calcium co-administration did not reach statistical significance. The majority of the evidence is derived from trials enrolling elderly women.

#### Vitamin D and muscle strength or function

We identified two SLRs (48, 49) that included the effects of vitamin D on muscle strength, which are presented in the summary table 10.

Stockton et al. (48) included 17 studies. Inclusion criteria included randomized RCTs involving adults, who were older than 18 years of age. The quality of the studies was assessed on the PEDro scale and a median score of 8 out of 10 (range 4–10; mode 8) was found. The trials used a variety of vitamin D supplementation regimes. Six trials compared vitamin D alone with placebo, four of which used vitamin D_2_, and two used vitamin D_3_. One study compared 1,25(OH)_2_D with placebo. Treatment with a combination of vitamin D_3_ and calcium supplements was used in nine studies. Five studies compared vitamin D and calcium with calcium alone, three studies investigated calcium and vitamin D versus placebo and one study used calcium and vitamin D versus nothing. Finally, one study investigated vitamin D via sunlight exposure (with a clearly defined exposed region and a documented daily exposure time) to usual care. Two studies did not state baseline 25(OH)D concentration, participants in four studies had baseline 25(OH)D >50 nmol/l, the mean baseline 25(OH)D level was 25–50 nmol/l in seven studies, and <25 nmol/l in four studies.

Meta-analysis showed no significant effect of vitamin D supplementation on grip strength or proximal lower limb strength in adults with 25(OH)D concentrations >25 nmol/l. Pooled data from two studies in vitamin D deficient participants (25(OH)D <25 nmol/l) demonstrated a large effect of vitamin D supplementation on hip muscle strength. *The Authors′ conclusions were*

vitamin D supplementation does not have a significant effect on muscle strength in adults with baseline 25(OH)D >25 nmol/L. However, a limited number of studies demonstrate an increase in proximal muscle strength in adults with vitamin D deficiency.

Muir et al. (49) included 13 RCTs, of which 8 were included in the Stockton analyses (48). The authors focused on the relation between vitamin D and balance, gait, and muscle strength as outcomes. The average age of the subjects in the studies was 78±4.1 years.

Statistically significant improvements in physical performance were noted in nine studies. Only one study demonstrated a beneficial effect on balance of a single large dose of vitamin D. All studies with daily doses of 20–25 µg demonstrated beneficial effects on balance and lower extremity muscle strength. The same vitamin D doses had beneficial effects in the two general populations of community-dwelling and older adults in institutional dwellings. Six of the eight studies that showed a beneficial neuromuscular effect included calcium supplementation in the regimens.

Twelve of the 13 RCTs included in this systematic review reported mean serum 25(OH)D concentration at baseline. Ten of these were in the deficiency range (<50 nmol/l) and two studies in the insufficiency range (50–75 nmol/l). Ten studies reported mean serum 25(OH)D concentrations at the end of the intervention period. In the intervention groups, three studies reached normal 25(OH)D concentrations with vitamin D supplementation and achieved improvements in muscle strength, gait, or balance function. Six studies showed an increase from <50 nmol to >50 nmol but <75 nmol/l after intervention, and four demonstrated a significant positive effect on physical function. One study was not able to improve the low 25(OH)D concentrations with treatment and did not demonstrate a positive effect on physical function outcomes. Statistically significant improvements in physical performance were noted in nine studies. Only one study demonstrated a beneficial effect on balance of a single large dose of vitamin D. All studies with doses of 20–25 µg/day demonstrated beneficial effects on balance and lower extremity muscle strength. Vitamin D doses of 20–25 µg had beneficial effects in the two general populations of community-dwelling and institutional-dwelling older adults. Six of the eight studies that showed a beneficial neuromuscular effect included calcium supplementation in the regimens.

Meta-analysis was performed for the outcomes of balance (body sway, Timed Up and Go (TUG) test), lower extremity muscle strength (knee extension), and grip strength without stratification according to dose or treatment regimen. The summary standardized mean difference, derived from studies with a total of 207 participants, on postural sway indicating a reduction in sway. Three studies with a total of 274 participants showed a decrease in time to complete the TUG test. A positive gain in knee extension strength was found.

Muir et al. (49) concluded that

vitamin D supplementation in doses of 20 µg to 25 µg/d have a beneficial effect on balance and muscle strength. An effect on gait was not found, although the studies that evaluated gait were of lower methodological quality and used low doses of vitamin D.

#### Vitamin D and cancer

We identified four SLRs that meet our inclusion criteria (28, 50–52) regarding vitamin D and cancer.

Total cancer. Two of the identified SLRs presented data on the relationship between vitamin D and total cancer (28, 51). Details of the SLRs are given in summary table 11.

In the report by Chung al. (28), two B-graded RCTs in addition to two B- and C-graded cohort studies were included. The findings were sorted by some lifestage groups, that is, 19–50, 51–70, and ≥71 years, in addition to postmenopausal women. None of the included studies showed significant relationships between either total cancer and serum 25 (OH) D concentrations (the cohort studies) or supplement intakes (the RCTs, 25 µg/day or 2,500 µg/month). No gender interaction was found.

The IARC report (51) included three original papers on serum 25(OH)D concentrations and total cancer mortality. No scientific quality of the studies was included. These were all cohort studies of which one found no significant relationship between 25(OH)D concentration and total cancer whereas another study found a significant twofold increased risk for cancer deaths in subjects with 25(OH) D concentrations below 37.5 nmol/l. The third cohort found that an increment of 25 nmol/l was significantly associated with 17% reduction in total cancer incidence and 29% reduction in cancer mortality.

#### Colon/colorectal cancers

Colon or colorectal cancers were included in all four of the identified SLRs (28, 50–52) and are summarized in summary table 12. The report by the World Cancer Research Fund (50) concluded that

the evidence on vitamin D was inconsistent and stated that there is limited evidence suggesting that foods containing vitamin D, or better vitamin D status, protect against colorectal cancer.

The evidence for a protective effect of intakes of food containing vitamin D and colorectal cancer was therefore rated as *limited suggestive*.

The overall conclusion in the IARC report (51) on vitamin D and colorectal cancer was that the observational evidence for an inverse association between serum 25(OH)D concentrations was *consistent* and *persuasive*, however evidence for a causal link is limited due to possible confounding which is not controlled for. No scientific quality of the studies was included. The report states in the overall conclusion, RCTs have ‘not demonstrated an effect of vitamin D supplementation on colorectal cancer risk, but due to several issues (doses, interaction, duration), they cannot be judged as contradictory to the evidence from observational studies either’.

Chung et al. (28) identified one B-rated RCT, one B-rated cohort study, and five B-rated and two C-rated nested case-control studies on the relationship between vitamin D and colorectal cancers. The RCT was based on the relationship between vitamin D_3_ supplementation and cancer and was conducted among the elderly. This study reported negative results for supplemental vitamin D_3_ versus no supplements. The B-rated cohort study included in the Chung et al. report (28), found a reduced risk of colorectal cancer associated with higher concentrations of 25(OH)D, that is, concentrations <50 nmol/l as reference gave adjusted OR at 0.44 (0.20–0.95) and 0.28 (0.11–0.68) for levels 50–80 and ≥80 nmol/l, respectively. Chung et al. (28) reported that most nested case-control studies found no significant associations between 25(OH)D concentrations and risk of colorectal cancer incidence or mortality, except for two of the three B-rated nested case-control studies in women, where statistically significant trends between higher 25(OH)D concentrations and lower risk of colorectal cancer were found. However, no individual quartile of 25(OH)D concentration had a significantly increased risk of colorectal cancer when compared to the reference quartile.

In the SLR by Yin et al. (52), one cohort study was included and seven nested case-controls. No scientific quality of the studies was included. The authors’ conclusion was that

the results support that serum 25(OH)D concentration is inversely related to colorectal cancer risk.

Breast cancer. Breast cancer was included in three of the identified SLRs (28, 50) (51). Details of the SLRs are presented in summary table 13.

The overall conclusion in the IARC report (51) suggest

observational evidence of an inverse association between 25(OH)D and breast cancer, however, the overall evidence is weak when case-control are not included in the meta-analysis and the heterogeneity between studies are large.

No scientific quality of the studies was included.

The Chung et al. (28) report included one B-rated cohort study and two B-rated nested-case control studies on the relationship between vitamin D and breast cancer. The report concluded that studies on vitamin D intake and risk of breast cancer were generally negative and points out that studies on 25(OH)D concentrations and breast cancer risk were very heterogeneous. Chung et al. (28) concluded that meta-analysis showed a non-significant protective effect on 25(OH)D concentration in blood and breast cancer, but based on very heterogeneous results. One cohort study (NHANES III) assessed 25(OH)D concentrations and the risk of breast-cancer-specific mortality, and found a significant decrease in breast-cancer-specific mortality during 9 years of follow-up in those with 25(OH)D concentrations >62 nmol/l. However, the nested case-control studies did not find a relationship between 25(OH)D and risk for breast cancer (7–12 years follow-up time).

The World Cancer Research Fund report (50) evaluated both post- and pre-menopausal breast cancer in relation to vitamin D exposure. However, the data were either of too low quality, too inconsistent, or the number of studies too few to allow conclusions to be reached.

Prostate cancer. Prostate cancer was included in three of the identified SLRs (28, 50) (51). Details of the SLRs are presented in summary table 14.

The Chung et al. report (28) identified 12 studies, all nested-case control studies, on the relationship between vitamin D and prostate cancer in 14 publications. Three of these were rated B and the rest was rated C in scientific quality. Ten publications reported no relationship between 25(OH)D concentrations and prostate risk. One study, rated C, found an increased risk associated with the lowest quartile compared with the highest quartile (<30 compared to >55 nmol/l). This study also found an increased risk for prostate cancer in men <52 years but not for the ≥51 years old with ≤ 40 nmol/l 25(OH)D concentrations compared with >40 nmol/l. A later publication, rated C, based on men from the Nordic countries showed a U-shaped relationship between 25(OH)D concentrations and prostate cancer risk.

The IARC report (51) concluded that *‘*observational studies have provided evidence for little or no effect of vitamin D and prostate cancer’. Moreover, the World Cancer Research Fund report (WCRF, 50) evaluating vitamin D and prostate cancer concluded that

data were either of too low quality, too inconsistent, or the number of studies too few to allow conclusions to be reached.

#### Vitamin D and diabetes

Diabetes type 1. One SLR was identified on the relationship between vitamin D and diabetes type 1 (53). Five studies were included, that is, one cohort study rated B and four case-controls rated B. The overall conclusion in their work was that

supplementation with vitamin D in early childhood may offer protection against diabetes type 1, however, randomized controlled trials are needed to establish causality.

Details for this SLR are given in summary table 15.

Diabetes type 2. We identified two systematic reviews (54, 55) and one RCT (56) on the relationship between vitamin D and risk for diabetes type 2. The papers are summarized in summary table 16.

Parker et al. (54) included nine studies in their SLR and meta-analysis. No grading of scientific quality of the included studies was given. They found an overall decrease in the prevalence of diabetes associated with higher 25-(OH)D concentrations. The conclusion in their work was that ‘high levels of vitamin D were associated with substantial decreased risk of diabetes type 2 and that further controlled trails are needed to evaluate causal associations’.

Pittas et al. (55) included four studies (two graded as fair quality and two as good) based on three cohorts. In addition, eight RCTs, three graded as good and five as fair, were included on this SLR. Pittas et al. (55) concluded that

the relationship between vitamin D and diabetes type 2 remains uncertain and that trials showed no clinical significant effect of vitamin D supplementation at the dosages given.

The RCT (56) did not support a protective effect of 20 µg vitamin D/day on diabetes type 2. We rated this as grade A in scientific quality. Diabetes was, however, not the primary outcome in this study.

#### Vitamin D and multiple sclerosis

No SLR on the relationship between vitamin D and multiple sclerosis for the general healthy population was identified in our search or in the additional search on recent RCTs.

#### Vitamin D and body weight

We only found one SLR (28) reviewing the relation between vitamin D and body weight. Three RCTs intervening with vitamin D alone were identified, and no effect on body weight was found. Chung et al. (28) also reviewed two RCTs intervening with vitamin D and calcium. In the Women's Health Initiative (methodological quality rate B) intervening with 10-µg vitamin D and 1,000-mg calcium (about 36,000 subjects) over 7 years lead to a statistical but not clinical significant effect (the weight change was 0.13 kg lower in the intervention group). In the other, and much smaller RCT (*n*=63), the intervention group (10-µg vitamin D and 1,200-mg calcium daily) lost 1 kg more over 15 weeks than the control group, but the difference was not statistically significant (methodological quality rate C). For details see summary table 17.

#### Vitamin D and total mortality

We identified three SLRs that met our inclusion criteria (27, 35, 54). Please refer to summary table 18 for details.

In the Cochrane review by Avenell on vitamin D and fractures (35), they also assessed the effect of interventions with vitamin D on deaths (total mortality) as a secondary endpoint. Based on 23 trials, the risk ratio (RR) of death was 0.97 (95% CI 0.93–1.01) in those given vitamin D with or without calcium compared to those given placebo or calcium. However, in those given vitamin D plus calcium versus placebo or control (14 trials, 54,203 participants), the RR of death was 0.94 (95% CI 0.89–0.99).

The Cranney et al. report (27) concluded that data from four cohorts suggest no association between baseline 25(OH)D concentrations and total mortality, but one cohort reported a statistically significant inverse trend. In meta-analyses including four RCTs, (13,899 participants, supplementation with vitamin D alone) had no significant effect on all-cause mortality (RR 0.97, 95% CI 0.92–1.02), and neither did supplementation with vitamin D and calcium (11 trials, 44,688 persons) result in a significant reduced mortality (RR 0.93, 95% CI 0.86–1.0) although the point estimate was very similar to that found in Avenell et al. (35).

A Cochrane review (57) reported that intervention with D_3_ with or without calcium versus placebo or no intervention (32 studies, 74,789 participants) resulted in a 6% reduction in total mortality (RR 0.94, 95% CI 0.91–0.98). The effect was only significant in trials giving vitamin D_3_ in combination with calcium. However, D_3_ alone was merely tested out in a quarter of the trials, and the difference between trials intervening with vitamin D_3_ alone and trials intervening with vitamin D_3_ and calcium was not significant. Significant effects were found in trials including participants with low vitamin D status (<50 nmol/l) and in studies intervening with daily doses lower than 20 µg. However, the differences from the other trials (vitamin D adequacy (RR = 0.92, 95% CI 0.7–1.07) and dose ≥20 µg, respectively (RR = 0.96, 95% CI 0.92–1.01) were not statistically significant. Vitamin D_2_ did not reduce mortality.

In the discussion, Bjelakovic et al. (57) also refer to a Swedish cohort study among elderly men. In this study, both low 25(OH)D-concentrations (<46 nmol/l, 10% of the men) and high concentrations (>98 nmol/l, 5% of the men) were associated with increased all-cause mortality (58).

#### Vitamin D and hypertension/blood pressure

Four SLRs (28, 55, 59, 60) and one RCT (61) on blood pressure or hypertension outcomes fulfilled our selection criteria, see summary table 19 for details.

For hypertension, Chung et al. (28) assessed a combined nested case-control study of men from Health Professional follow-up study (HPFS) and women from Nurses’ Health study (NHS). This analysis showed a fivefold incidence of hypertension in men after 4 and 8 years who had 25(OH)D-concentrations below 37.5 nmol/l at baseline compared with those above 37.5, and sixfold higher than those above 75 nmol/l. Women with 25(OH)D below 37.5 at baseline had also higher incidence of hypertension after 4 years but not 8 years. A nested case-control study from the NHS2 showed that after 7 years, women in the three quartiles with baseline values below 80.5 nmol/l were 50–60% more likely to develop hypertension than those in the highest quartile. Chung et al. (28) also evaluated the relationship between combined vitamin D and calcium. The only study that was included was the Women's Health Initiative and they did not find any effect of vitamin D and calcium on the risk of hypertension.

For blood pressure, Chung et al. (28) included three trials, one British (grade A), one German (grade B), and one Indian study (grade B) with different doses of vitamin D (20 µg daily, a single dose of 2,500 or 3,000 µg every 2 weeks) compared with placebo None of the studies reported significant differences in diastolic blood pressure, while systolic blood pressure was decreased by 6 mm Hg in one study of older women who received both 20 µg vitamin D and calcium compared with calcium alone. The study of British older adults showed no effect of a single dose of 2,500 µg compared with placebo. In both study arms, systolic and diastolic blood pressure decreased to a similar extent. The Indian study of obese men (6 weeks of vitamin D, 3,000 µg every 2 weeks) reported a close to statistically significant increase in systolic blood pressure in the intervention group.

Witham et al. (59) included 11 RCTs, 3 of which used vitamin D_3_ or vitamin D_2_ as intervention in hypertensive adults. The studies were reported to be of variable quality. Two of the studies were included in Chung et al. (28). Four studies used either 1,25-(OH)2D or synthetic 1-α-calcidiol, and one UVB-irradiation. Eight studies were performed in hypertensive patients. Two studies used vitamin D_3_ in normotensive subjects and no effect of supplementation was seen. The authors conclude: ‘We found weak evidence to support a small [lowering] effect of vitamin D on blood pressure in studies of hypertensive patients’.

Wu et al. (60) assessed four RCTs with changes in blood pressure as outcome, two of which were included in the Chung et al. review (28). The quality of the studies was assessed but not reported. One of the additional studies used 5-µg vitamin D_3_ and the other 10-µg vitamin D_3_ with calcium compared with placebo. They conclude:

Oral vitamin D supplementation may lead to a reduction in systolic blood pressure but not diastolic blood pressure. Given the small number of trials and small but statistically significant reduction in blood pressure, further studies and required to confirm the magnitude of the effect of vitamin D on blood pressure reduction and define optimum dose, dosing interval, and type of vitamin D to administer.

Pittas et al. (55) conducted a systematic review and meta-analysis on cardiometabolic outcomes and vitamin D, including 32 studies on diabetes, hypertension, and blood pressure. The three cohort studies assessing hypertension risk were all included in the Chung et al. report, showing significant associations between lower 25(OH)D concentrations and increased risk of hypertension. In addition, 10 RCTs of vitamin D supplementation were assessed in the review. Of these, three trials were considered of good quality, five of fair quality, and two of poor quality. Three studies not included in any of the above-mentioned systematic reviews, were assessed in this review, and two of those were rated as good quality. The Women‘s Health Initiative, rated good, combined a low-dose vitamin D supplement (10 µg/day) with calcium carbonate, the number of participants being about 36,000. This study found no effect on self-reported incident hypertension after 7 years of follow-up while a sub-group analysis found an increased risk of incident hypertension among black participants taking the supplements. However, vitamin D supplement alone was not assessed in this trial. The two additional trials included in this evaluation and not included in former reviews reported no significant effects of vitamin D supplements on blood pressure. Neither did the effect on systolic blood pressure differ between those trials providing higher (>25 µg vitamin D/day) or lower (<25 µg/day) doses. The authors concluded

A lower 25(OH)D concentration or vitamin D intake may be associated with higher risk of incident hypertension and cardiovascular disease.

We included one RCT (61), graded C, which was not included in the SLRs. The subjects were randomized to receive either 1,000 µg vitamin D_3_/week, 500 µg/week, or placebo. All subjects were given 500-mg calcium daily. No beneficial effects of the supplements were observed on blood pressure, while the group receiving 500 µg showed a slight but significant increase in systolic blood pressure compared with the placebo group. Mean baseline concentrations of 25(OH)D as well as blood pressure were within normal range in these subjects, and serum 25(OH)D concentration increased from 58 to 140 and 101 nmol/l in the two intervention groups. The authors’ conclusion was

Our results do not support a positive effect of vitamin D on hypertension. Further studies in subjects with low serum 25(OH)D levels combined with hypertension are needed.

#### Vitamin D and CVD clinical outcomes

Three SLRs of CVD outcomes and serum concentrations of 25(OH)D met our selection qualifications. These were Chung et al. (28), Parker et al. (54), and Grandi et al. (62). A fourth systematic review, Wang et al. (63), focused on vitamin D supplementation and CVD and one further review by Pittas et al. (55) analyzed cardiometabolic outcomes and vitamin D concentrations. See summary table 20.

Chung et al. (28) focused on vitamin D and CVD. They included one RCT, one nested case-control study and one cohort study. The RCT, where almost 2,700 elderly British community-dwelling men and women received either placebo or 2,500-µg vitamin D every 4 months, reported no significant effects on total cardiovascular deaths, ischemic heart disease, myocardial infarction, or stroke after 5 years. Still there were fewer cardiovascular deaths (RR = 0.84; CI 0.56–1.10) as well as ischemic heart disease deaths (RR = 0.84; CI 0.56–1.27) in the intervention group receiving vitamin D than in the placebo group. In contrast, both cohort studies showed significantly lower risk associated with increased serum concentrations of 25(OH)D. In the Framingham Offspring Study (FOS), men and women with serum 25(OH)D concentrations below 37.5 nmol/l were 50–70% more likely to have a cardiovascular event within the 5.4-year study period, compared with those with levels between 25 and 37.5 nmol/l while a multivariate analysis suggested an increased likelihood of cardiovascular events in people with S-25(OH)D below approximately 50 nmol/l. Similarly, the nested case control Health Professional follow-up study (HPFS) reported over two-fold risk in men with 25(OH)D below 37.5 nmol/l and a 60% increased risk of cardiovascular events in those with concentrations between 56 and 75 nmol/l compared with those above 75 nmol/l or higher.

Grandi et al. (62) evaluated the prognostic value of 25-OH-D concentrations for CVD incidence and mortality. They reviewed seven prospective studies in addition to the two included in the Chung et al. (28) report. Three out of five mortality studies reported significant associations, with one showing a fivefold increase in risk in those with concentrations in the lowest quartile, below 30.7 nmol/l. However, two large population-based studies reported no significant effect on cardiovascular mortality. Mean age was lower in these two studies, or 44.8 and 49.4 years, respectively, compared with the studies reporting increased risk, where mean age ranged from 62 to 74 years. Grandi et al. included two additional incidence studies not included in the Chung et al. report, a New Zealand study of 1,471 postmenopausal women participating in a calcium supplementation trial, and a Finnish study of 689 men and women who were followed for up to 10 years. Neither study demonstrated a significant association of S-25(OH)D concentrations with cardiovascular events. Grandi et al. concluded

Data from prospective studies suggest an inverse relationship between 25(OH)D and cardiovascular risk. However, given the heterogeneity and small number of longitudinal studies, more research is needed to corroborate a potential prognostic value of 25(OH)D for cardiovascular disease incidence and mortality.

Parker et al. (54) evaluated the association between 25(OH)D and the presence of cardiometabolic disorders including CVD, diabetes, and metabolic syndrome. They reviewed and meta-analyzed 28 cross-sectional studies, case-control, cohort and RCTs on cardiometabolic disorders, including 16 studies with cardiovascular event outcomes and S-35(OH)D concentrations. Two of the seven additional studies of CVD outcomes not included in the review by Grandi et al. (62) did not report a significant association between CVD risk and vitamin D, four studies showed lower risk and one study of 216 people living in Southern India showed a 60% higher risk associated with higher S-25(OH)D concentrations. Meta-analysis of these 16 studies was consistent with *a 33% reduction in the risk of having a cardiovascular disease*. The authors conclude that

Our findings suggest that high levels of vitamin D, among adult populations, are associated with a substantial decrease in cardiovascular disease, type 2 diabetes and metabolic syndrome. Interventions targeting a positive modification of vitamin D deficiency in adult and elderly populations would substantially contribute to halting the current epidemics of cardio-metabolic disorders. Further controlled trials are needed to evaluate the causal association between vitamin D levels and cardio-metabolic disorders.

The systematic review of Wang et al. (63) assessed 17 studies in total out of which 6 were prospective studies and 4 were interventions on vitamin D with or without calcium supplementation for the risk of cardiovascular event outcomes. The quality was assessed but not reported in the article. None of the four RCTs on vitamin D supplements were specifically designed for CVD outcome, and five of the studies were on patients receiving dialysis. All five studies on haemodialysis patients showed a lower risk of cardiovascular events in those receiving vitamin D as did the single study on vitamin D in the general population. That study assessed the vitamin D intake and identified CVD endpoints. Supplemental intake greater than 20 µg/day was associated with a non-significant lower risk of CHD mortality RR= 0.80 (CI 0.57–1.13). The conclusions of Wang et al. (63) were:

Evidence from limited data suggests that vitamin D supplements at moderate to high doses may reduce CVD risk. Further research is needed to elucidate the role of these supplements in CVD prevention.

The systematic review of Pittas et al. (55) on vitamin D and cardiometabolic outcomes is an expansion of the evidence report for the Institute of Medicine decisions on vitamin D reference intakes (28). They included 32 studies on diabetes, hypertension, and blood pressure associated with either vitamin D status or intake of supplements. The quality was assessed but not reported in the article. These outcomes are evaluated separately below. Nine cohort studies analyzing CVD outcomes were also included in the review, seven of these were rated of good quality. Cardiovascular endpoints included myocardial infarction, cardiovascular-related death, a composite cardiovascular endpoint and stroke. All studies measured 25(OH)D concentrations. Overall, five out of the nine studies found that lower 25(OH)D concentrations were associated with increased risk for incident CVD. Five trials on vitamin D supplementation were included in the review. None of these reported a statistically significant effect of vitamin D supplementation on various cardiovascular outcomes, including myocardial infarction and stroke. The authors’ conclusion was:

The association between vitamin D status and cardiometabolic outcomes is uncertain. Trials showed no clinically significant effect of vitamin D in the dosages given. Adequate randomized controlled trials, conducted in well-defined populations, are needed to test the potential role of vitamin D in primary prevention or therapy. Vitamin D remains a promising, although unproven, new element in the prevention or management of cardiometabolic disease.

#### Vitamin D and infections

We identified three systematic reviews (28, 64) (65) and one additional RCT (66) related to vitamin D and infections that met our selection criteria (see summary table 21).

Chung et al. (28) assessed a single cohort study from the NHANES III on infectious disease mortality, stratified by baseline 25(OH)D concentration. No differences in infectious disease mortality were detected between quartiles ranging from <44 to >80 nmol/l after 7–8 years of follow-up.

Yamshchikov et al. (64) assessed 13 trials, 10 of which were placebo controlled, studying vitamin D for the prevention or treatment of infectious disease (bacterial, viral, and parasitical). The included clinical trials demonstrated substantial heterogeneity in patient demographics and vitamin D interventions. On the basis of these heterogeneous studies, the authors conclude:

More rigorously designed clinical trials are needed for further evaluation of the relationship between vitamin D status and immune response to infection.

Another systematic review and meta-analysis on S-25(OH)D concentrations and tuberculosis assessed seven observational studies, only one of which was in a European setting (65). All but one of the studies reported a significant association between low S-25(OH)D and active tuberculosis. The authors conclude:

Low serum vitamin D levels are associated with higher risk of active tuberculosis. Although more prospective studies are needed to firmly establish the direction of this association, it is more likely that low body vitamin D levels increase the risk for active tuberculosis.

Urashima et al. (66) published a double-blinded RCT of vitamin D supplementation to prevent influenza A in Japanese school children. This 4-month trial where 430 children aged 6–15 years were randomized into two groups, placebo or supplements of 30 µg/day, had influenza A, diagnosed by medical doctors using a rapid influenza diagnostic test (RIDT), as primary outcome. A reduced risk of influenza was observed in the group receiving 30-µg vitamin D daily, and more prominent in those who had not been taking other vitamin D supplements (RR = 0.36; 0.17–0.78). Influenza A occurred in 18 out of 167 children taking vitamin D, but in 31 of 167 in the control group. No difference was observed in influenza B. The study was rated C, in part as 25(OH)D concentrations were not measured.

### The effect of sun or UVB exposure on different outcomes in different population and age groups (Research question 5)

We identified one SLR on the relationship between both solar and artificial UVB radiation and 25(OH) in blood (27) (summary table 22). The report was based on eight RCTs. The overall quality of the trials was rated as low.

This SLR concluded that “there is fair evidence that solar and artificial UV-B exposure increase 25(OH)D levels. The included trials did not address the issue of whether serum 25(OH)D response is attenuated in heavily pigmented groups. It was also not possible, to evaluate the impact of effect modifiers such as age, ethnicity, seasonality and latitude.

The authors expressed that further research is needed to clarify the exact doses needed to maintain 25(OH)D concentrations over time, in the absence of supplementation.

### The UL for vitamin D for different health outcomes in different population and age groups (Research question 6)

Both Cranney et al. (27) and Chung et al. (28) included research questions related to this question. See summary table 23 for details.

Cranney et al. (27) performed an SLR of a total of 22 RCTs to answer if vitamin D supplementation resulted in toxicity. A total of 22 trials reported data on toxicity-related outcomes, 21 of which used doses above 10 µg/day. Only 12 received a rating of >3 on the Jadad scale (26). An adequate description of allocation concealment was reported in three trials. Toxicity results from trials with intakes of vitamin D above current reference intakes varied and this may have been related to different doses, baseline characteristics of populations or exposure times. Most trials excluded subjects with renal insufficiency or hypercalcaemia, were of small sample size and had short durations of exposure to vitamin D. Event rates were low across trials in both the treatment and placebo arms. The Womens′ Health Initiative trial in women aged 50–79 years, examined the effect of vitamin D_3_ 10 µg in combination with 1,000-mg calcium carbonate versus placebo and found an increase in the risk of renal stones corresponding to 5.7 events per 10,000 person-years of exposure The results are complicated by the fact that the subjects (intervention and placebo) were allowed to take additional vitamin D supplements up to 15 µg and later 25 µg per day and also calcium supplements up to 1,000 mg.

The authors conclude

that overall, there is fair evidence that vitamin D supplementation above current reference intakes, with or without calcium supplementation, was well tolerated. A significant increase in kidney stones was observed in one large trial in postmenopausal women taking 10 µg vitamin D3 with calcium. The quality of reporting of toxicity outcomes was inadequate in a number of the trials, and most trials were not adequately powered to detect adverse events.

Chung et al. (28) considered all the RCTs included in their SLR focusing on a number of health outcomes. Only 16 out of 63 RCTs reported adverse effects and they were generally not powered to detect them. Eleven of these reported at least one adverse effect.

According to Chung et al. (28), RCTs of vitamin D (doses ranged from 10 to 143 µg, 5,714 IU/day vitamin D_3_ or from 125 to 250 µg vitamin D_2_) and/or calcium supplementations (doses ranged from 200 to 1,500 mg/day) reported few cases of gastrointestinal disruption such as constipation, diarrhea, upset stomach, musculoskeletal soreness, primary hyperparathyroidism, hypercalcemia, renal calculi, and craniotabes. However, comparisons between the intervention groups and the control groups were not usually reported. One RCT reported some adverse events that required hospital admission, including retrosternal pain, a non-ST elevation myocardial infarction and a transient ischemic attack (all three cases in vitamin D 20 µg/day plus exercise training group) and one case of acute cholecystitis (in calcium, vitamin D plus exercise training group). Another RCT reported that ‘there were no significant differences between the vitamin D and the control groups in the rate of incident cancer and vascular disease (ischemic heart disease and stroke)’ (actual data not provided), and one participant died during the study. However, these adverse events may or may not be associated with vitamin D and/or calcium supplementation in this study.

We have found the following harms reported in some of the SLRs included in our review. Michael et al. (46) concluded that on the basis of the nine fair-quality trials related to falls included in their review, they found no increase in falls, fallers, or other major adverse events. Only three trials specifically reported adverse effects – transient and asymptomatic hypercalciuria or hypercalcaemia in the intervention group – but no differences in adverse effects or clinically significant harms, such as incident kidney stones, cancer, ischemic heart disease, or stroke. Gillespie et al. (45) found that adverse effects (hypercalcaemia, renal disease, gastrointestinal effects) were reported in three trials but none were statistically significant. One RCT study (38) reported an increased risk of fracture and falls in those elderly that were given a single yearly dose of vitamin D corresponding to about 18 µg/day.

Bjelakovic et al. (57) reported that vitamin D_3_ combined with calcium increased the risk of kidney stones (RR 1.17, 95% CI 1.02–1.34), whereas the effect of vitamin D was not significant on other side effects.

### The interactions of vitamin D with calcium intake on different health outcomes in different population and age groups (Research question 7)

In general, we were not able to distinguish vitamin D and vitamin D together with calcium in our systematic reviews. Thus, this question has been handled within the other research questions.

### The interaction of vitamin D intake or vitamin D status with vitamin A intake or vitamin A status on health outcomes in different population and age groups (Research question 8)

We did not find any SLRs on this topic.

## Discussion

The aim of this systematic review was to provide a scientific base for a Nordic recommendation for dietary intake of vitamin D. We analyzed the literature on the relationships between vitamin D, 25(OH)D concentration and different health outcomes. Moreover, we studied the relationship between vitamin D intake and 25(OH)D concentration. We focused on published systematic reviews but included a few RCTs which were published after the SLRs. Some of the SLRs included both observational studies as well as RCTs. In the result section, we did not include the recent American IoM report on vitamin D and calcium intake from 2010 (29) as it is not a systematic review. However, we included the SLRs forming the basis for the IOM report. We focused on populations in Europe and North America. However, if other populations were included in the SLRs, we were generally unable to separate them.

There are some general challenges when reviewing to establish evidence for the relationship between vitamin D and health. First, agreement has not yet been achieved for what is considered an optimal 25(OH)D concentration, second the relationship between 25(OH)D and health outcomes are likely to be confounded by diet, in particular fish intakes, but also physical activity, both of which are not easily adjusted for in observational studies. Third, in experimental studies vitamin D and calcium supplements are often combined, thus the separate effect of vitamin D supplements can be questioned.

### Assessment of vitamin D status

The reliability of the assays for serum/plasma 25(OH)D measurement has been questioned.

It has been shown in a number of studies that different assays give different results (e.g. (67–70)). In a recent Swedish study (71), the same samples were analyzed in three different laboratories. The results showed a large discrepancy in the concentrations. Thus, it seems fairly challenging to use the serum 25(OH)D-concentration as an outcome marker for assessing vitamin D deficiency and insufficiency as well as using it as an indicator of exposure.

### Role of UV exposure

Vitamin D is produced in human skin when exposed to the sun. It is the ultraviolet (UV) radiation in the UV-B brand, that is, wavelengths between 290 and 315 nm that are needed for the photo conversion of provitamin D_3_ to previtamin D_3_ to occur in the skin.

At latitudes above ∼50°N, both the qualitative and quantitative properties of sunlight is not sufficient in parts of the year for vitamin D production to take place (72), leading to the so-called vitamin D winter. In Copenhagen, the vitamin D winter is estimated to start in mid-November and last until end of February whereas in Tromsø it is estimated to last 2.5 months longer. In Helsinki (60°N), the length of the vitamin D winter spans from mid-October to mid-March (73) and in Reykjavik (64°N) from early October to late March. In addition to latitude and season, the actual vitamin D production in skin in humans is affected by several individual and external factors. The ozone layer effectively absorbs UVB light, and clouds, when completely overcast, can attenuate the UVB radiation as much as 99%. Surface reflection, especially from snow can however reflect the UVB radiation up to 95%.

Time spent outdoors, the use of sunscreen, and clothing also affect the sun-induced vitamin D for individuals (74). In addition, individual vitamin D status has been shown to affect the effectiveness of cutaneous vitamin D_3_ production, so that individuals with initial low levels of 25(OH)D seem to have a lower threshold concentration for vitamin D production in skin compared to individuals with higher concentrations (75). The sun-induced vitamin D production can be up to six times higher in people with pale skin compared to people with dark skin (76). The skins ability to produce vitamin D also decreases with age (77).

A down-regulating mechanism of vitamin D production in skin prevents vitamin D toxicity due to prolonged sun exposure by a photo-degradation of previtamin D_3_ to biologically inert isomers (78).

Data available on seasonal variation in 25(OH)D concentrations in the general population in some Nordic countries have demonstrated less fluctuation between summer (79, 80) and winter compared to other comparable populations (81). The vitamin D intake in the diet and common use of supplements is a possible explanation for this (21). However, in studies from Finland a marked seasonal variation in S-25(OH)D concentrations has been observed in adolescents, in adults and in the elderly (41, 78) (82). A study from Sweden (83) among women aged 61–83 years, an increase from winter until summer in 25(OH)D concentrations was found to be 38%. In Iceland, adults who do not take vitamin D supplements show a marked seasonal variation (13). The magnitude of the difference in 25(OH)D concentrations between summer and winter decreases with increasing latitude (75).

### Main findings in relation to the research questions

#### What is the effect of vitamin D from different sources on serum 25-OHD concentrations? (Research question 1)

Our first question was related to the effect of vitamin D from different sources on serum 25(OH)D concentrations. We did not find any SLR on the effect of natural vitamin D sources on 25(OH)D concentration. However, we are aware of one study in which the effect of edible wild mushrooms (*Cantharellus tubiformis*) on 25-OHD-concentration has been studied (84). In that study, a portion containing 15 µg vitamin D_2_/day increased the 25-OHD concentration to the same extent as a corresponding supplement of vitamin D_2_, from about 30 to about 45 nmol/l over 3 weeks.

Regarding fortified foods and supplementation, the SLRs indicated that there is a clear effect of fortified foods and supplementation on the S-25OHD concentration. However, it is not easy to conclude what doses are needed to achieve specific levels of 25-OHD. One SLR (Black et al., 30) estimated that 1 µg ingested from fortified foods increased the S-25(OH)D concentration by 1.2 nmol/l. Two SLRS focused on supplementation (27, 32). In the SLR by Cashman et al. (32), the authors focused on studies performed at latitudes higher than 49°N, which is applicable for the Nordic countries. Both SLRs found a positive effect of vitamin D intake, including fortified foods (27) and supplements on 25-OHD concentrations. Cranney et al. (27) concluded that the meta-regression results suggested that 2.5 µg/day of vitamin D_3_ will increase the serum 25(OH)D concentrations by 1–2 nmol/l. This suggested that doses of 10–20 µg daily may be inadequate to prevent vitamin D deficiency in at-risk individuals. Vitamin D_3_ doses of 17.5 µg daily or more significantly and consistently decreased serum concentrations of PTH in vitamin-D-deficient populations. However, Cranney et al. (27) concluded that the increase in S-25(OH)D concentration was higher when the baseline concentration was low than when it was high, and also that the increase was larger if the duration of the study was longer. Cashman et al. (32) included age groups between 9 and 78 years. Using meta-regression analyses, they calculated that an average of 9 µg vitamin D was needed for a population to achieve 50 nmol/l. However, taking interindividual variation into account in the meta-regression analysis, 23.5-µg vitamin D/day was needed for 95% of the population to reach a serum level ≥50 nmol/l. However, the authors concluded that these latter results have to be treated with caution as the number of data points in the analysis is low.

Cranney et al. (27) considered different age groups. They concluded that 5-µg vitamin D/day may not be enough to prevent vitamin D deficiency in some infants at northern latitudes. A response in 25(OH)D concentration of vitamin D supplementation was seen in the trials included. Most of them used vitamin D_2_. Supplementation during pregnancy with either 25–90 µg/day vitamin D_2_ or 25 µg vitamin D3 increased 25(OH)D concentration both in the mothers and cord blood. Cranney et al. (27) included one study on supplementation during pregnancy (85), in which they did not find an effect of 25-µg vitamin D given to the mother on the infant S-25(OH)D concentration. The authors actually performed another 15 week trial in the winter months after that (86) and found that giving 50-µg vitamin D_3_/day to the lactating mothers increased the infants’ S-25(OH)D to almost the same level as 10-µg vitamin D_2_/day to the infant – the level being ca. 70 and 83 nmol/l respectively. This study was excluded from the Chung analyses as it was regarded not to be an RCT. Cranney et al. (27) identified four trials in children and adolescents and found consistent increases in S-25(OH)D concentrations with different doses: 8 nmol/l with 5-µg vitamin D_3_/day, 16.5 nmol/l with 15 µg/day and 60 nmol/l with 50 µg/day. In premenopausal and younger males, a dose effect was noted in those trials that used multiple doses of vitamin D_3_. In this age group, Cranney et al. (27) also compared the effect of vitamin D_2_ and D_3_ on S-25(OH)D concentration and concluded that vitamin D_2_ appeared to have a smaller effect than vitamin D_3_. A dose response was noted in trials in postmenopausal women, older men and in elderly populations in long-term care or nursing homes. The doses had a large range, the basal S-25(OH)D concentrations varied and the assays used were very heterogeneous.

#### What is the relationship between 25-OHD concentrations/dietary vitamin D intake/supplemental vitamin D and different outcomes in different population and age groups? (Research questions 2–4)

Three questions were related to the relationship between 25(OH)D-concentrations or dietary vitamin D or supplemental vitamin) and different health outcomes. In addition to RCTs, the SLRs included mostly cross-sectional, cohort or longitudinal studies. RCTs have been performed only for skeletal outcomes, falls, muscle function and weight and these have been included in the SLRs. In some cases, secondary analyses of the RCTs on some health outcomes have been performed. We found some evidence for a causal relationship with bone health, falls and muscle strength, and total mortality. We did not find evidence for establishing a causal relationship between vitamin D intake, vitamin D supplementation, or serum 25-OHD concentration and most other the health outcomes.

#### Pregnancy

De-Regil et al. (33) reviewed six randomized trials including 1,023 pregnant women. They concluded that there is currently insufficient high-quality evidence relating to the clinical effects of vitamin D supplementation during pregnancy. The other SLR (28) included only one nested case-control study reporting that a 25(OH)D concentration lower than 37.5 nmol/l was associated with an increased risk of preeclampsia.

#### Growth

Seven intervention studies and two observational studies were included in one SLR (28). The authors concluded that due to the lack of methodologically solid studies, they were cautious in their conclusions about the effect of vitamin D in newborns, infants, and children.

#### Skeletal effects

Low 25(OH)D concentration increases the risk of rickets. The threshold is uncertain, but a number of the studies suggest increased risk at S-25(OH)D concentrations <27.5 nmol/l. Many studies were conducted in developing countries with low dietary calcium intake. Low calcium intake may influence the relationship between 25(OH)D and rickets, and the 25(OH)D threshold for rickets in populations with high calcium intake is unclear. It could be added that vitamin D has been used as a prophylaxis in the Nordic countries for decades, and the current recommended daily dose of 10 µg seems to be effective in preventing rickets if the supplement is given (87).

The data on the relationship between vitamin D and *bone mineral content or bone mineral density are heterogeneous*. In infants, there is fair evidence for an inverse relationship between 25(OH)D and PTH at low concentrations, whereas the relationship between 25(OH)D and BMC is inconsistent. In older children and adolescents, there is fair evidence for 25(OH)D to be associated with change in BMC/BMD, but RCTs have not consistently shown an effect of vitamin D supplementation. A Cochrane review (34) in children found no overall effect of vitamin D supplementation, but an effect was suggested in populations with low concentrations of 25(OH)D. There was insufficient evidence for a relationship between 25(OH)D and change in BMD in pregnant women, and one good cohort study did not find any relationship between 25(OH)D and BMD during lactation. Based on observational studies, there is fair evidence for a relationship between 25(OH)D and BMD or change in BMD at the femoral neck in the elderly. From intervention studies it is good evidence that supplementation with vitamin D combined with calcium leads to a small increase in spine, femoral neck, total hip, and total body BMD. Based on available studies, it is less certain that vitamin D supplementation alone has an effect on BMD.

It is challenging to describe optimal concentration of 25(OH)D for bone mineral density or bone mineral content based on available SLRs. In infants, a threshold around 27 nmol/l might exist for the relationship between 25(OH)D and PTH (27). This threshold was reported as 30–83 nmol/l in older children and adolescents (27). The SLR by Cranney et al. (27) also refers to one RCT among adolescent girls reporting an effect of vitamin D intervention to occur at mean levels of 25(OH)D >50 nmol/l, whereas the Cochrane review by Winzenberg et al. (39) only reported an effect of vitamin D supplementation in children with low mean 25(OH)D at baseline (≤ 35 nmol/l). In observational studies among the elderly, the bone loss at the hip was increased at concentrations of 25(OH)D ranging from 30 to 80 nmol/l in different studies.

*Fracture* is the primary skeletal health outcome in adults. Based on available data, the SLRs concluded that intervention with vitamin D (dose 10–20 µg/day) combined with calcium reduces the risk of *total fracture and hip fracture*, whereas intervention with vitamin D alone has not shown an effect in the doses tested out. Although a threshold of 74 nmol/l was considered to show a reduction in total fracture incidence, the variability in analytical methods and the fact that S-25(OH)D was assayed only in subsamples, make this threshold unreliable. To what extent an intervention with vitamin D and calcium prevent fracture in non-institutional elderly is debated. Both the Cochrane review by Avenell et al. (35) and the Cranney et al. (27) report only found a significant effect in studies performed in institutionalized elderly, although the difference between studies performed in institutionalized and community dwellers was not statistically significant. However, the DIPART study (37) reported that the effect was found across a wide range of age (mean age 70 years; range 47–107 years).

Although the overall conclusion is that intervention with vitamin D *alone* in the doses tested has not been proven effective in preventing fractures, it could be added that a British study by Trivedi et al. (88), Jadad score 3 (26) reported that 2,500-µg vitamin D every 4th months (corresponding to 20 µg/day) compared to placebo, reduced the risk for any new fracture (OR 0.78 [95% CI 0.61–0.99]). On the other hand, the Australian RCT referred to previously, reported an increased risk of fracture in those given a single yearly high dose of vitamin D (38).

Currently, there is also interest in studying the effect of higher doses of vitamin D: Vital (ClinicalTrials.gov; NCT01169259), FIND (NCT01463813) and DO-Health(not registered as yet).

It could be added that a recent Swedish study referred to in the IOM report found increased risk of fracture in men with S-25(OH)D below 40 nmol/l (89).

#### Dental health

Lack of data precludes any conclusion concerning the relation between vitamin D and dental health.

#### Falls

Six SLRs were focused on vitamin D intake or 25(OH)D and falls. There was overall fair evidence that vitamin D with calcium is effective in preventing falls in the elderly especially in those with low baseline 25(OH)D concentrations, both community dwelling and in nursing care facilities. One SLR concluded that vitamin D was effective (43). Some but not all SLRs concluded that a dose greater than 20 µg was effective, in conjunction with calcium supplementation. One study suggested that 25(OH)D concentrations below 39 nmol/l were associated with an increased risk of falls.

#### Muscle function

Two SLRs focused on vitamin and outcomes related to muscle function in the elderly. Stockton et al. (48) concluded that vitamin D supplementation does not have an effect when basal 25(OH)D is greater than 25 nmol/l, but that vitamin D has an effect in adults with vitamin D deficiency. However, Muir et al. (49) concluded that vitamin D doses of 20– 25 µg/day have beneficial effects on balance and muscle without taking baseline S-25(OH)D into account. Thus, doses of 20–25 µg/day could be beneficial for muscle function in the elderly, but the information on the effect of lower doses was scarce.

#### Cancer

Vitamin D and cancer have been studied in a number of cohort studies. Some RCTs have been performed but they are secondary analyses of supplemental studies for the prevention of fractures (88, 90). There was not consistent evidence for an association between vitamin D status and total cancer in SLRs including cohort studies and RCTs. There is some observational evidence of an inverse association between vitamin D status and risk of colorectal cancer, however evidence for a causal relationship are lacking. The evidence for an inverse association between vitamin D status and breast cancer risk is weak due to lack of good quality studies and heterogeneity between studies. There is little or no evidence for a protective effect of vitamin D on prostate cancer.

#### Diabetes and multiple sclerosis

The evidence for a causal relationship or an association between vitamin D and type 1 and type 2 diabetes is limited and inconclusive. Lack of data precludes any conclusion concerning the relation between vitamin D and multiple sclerosis.

#### Body weight

There is no clear evidence for vitamin D to influence body weight development.

#### Total mortality

Based on the RCTs in the SLRs it is concluded that vitamin D3 (10–20 µg/day) combined with calcium significantly reduces total mortality. However, it is uncertain if co-supplementation with calcium is necessary to achieve this effect. It could be added that a recent a Swedish cohort study among elderly men followed for around 14 years reported increased all-cause mortality both in men at the low (<46 nmol/l) and the high end of 25(OH)D concentrations (>98 nmol/l) (58). Moreover, a recent Danish cohort study among subjects from the Copenhagen general practice sector including near to 250,000 subjects that were followed for 3 years found a reverse J-shaped relationship with the lowest mortality at 25(OH)D concentrations of 50–60 nmol/l (91).

#### Hypertension and blood pressure

Evidence from RCTs reviewed in four SLRs on blood pressure is inconclusive. Some RCTs detected a small reduction in diastolic blood pressure, particularly in people with higher baseline values, while another showed a small reduction in systolic pressure. All SLRs concluded that there was a need for further studies to explore this relationship for possible clinical significance. A recent RCT (61) which was not included in the SLRs did not support a positive effect of vitamin D supplementation in conjunction with calcium on hypertension, intervening with large doses (500 or 1,000 µg/week with 500-mg calcium) in overweight persons over 1 year.

However, low vitamin D status has repeatedly been associated with a higher incidence of hypertension as reviewed in two SLRs. Nested case control studies show marked reverse associations between incidence of hypertension and 25(OH)D in men and women with baseline <37.5 nmol/l compared with >37.5 nmol/l and also compared with those over 75 nmol/l.

#### Cardiovascular clinical outcomes

Systematic reviews based on cohorts or case-control studies have repeatedly found an association between low 25(OH)D concentrations, mostly below 37.5 or 50 nmol/l and an increased risk of CVD. However, a significant effect of supplementation on cardiovascular outcomes has not been reported. The trials in question were all designed for health outcomes other than CVD.

#### Vitamin D and infections

The evidence for an effect of vitamin D on infections is scarce and trials were very heterogeneous.

#### What is the effect of sun or UVB exposure on different outcomes in different population and age groups? (Research question 5)

The only SLR assessing this question concluded that there is fair evidence that both solar and artificial UV-B exposure increase 25(OH)D concentrations. We were not able to establish a dose response relationship. We did not find any SLR addressing the effect of sun or UVB exposure and other outcomes.

#### Which is the UL (Tolerable Upper Intake Level) for vitamin D for different health outcomes in different population and age groups? (Research question 6)

The SLRs did not give any definite answer to this question. Chung et al. (28), Cranney et al. (27), Vestergaard et al. (36), Avenell et al. (35) and Bjelakovic et al. (57) included adverse effects in their reviews of RCTs. Vitamin D given with calcium, but not vitamin D alone, moderately increased the risk of renal stones.

There are some observational studies suggesting that total mortality is increased at high 25(OH)D concentrations (58, 91). Some studies have reported an increase in prostate cancer (92) or total cancer (58) at higher 25(OH)D-concentrations. A trial using large yearly doses of vitamin D reported increased incidence in fractures and falls in the elderly (38).

#### Which are the interactions of vitamin D with calcium intake on different health outcomes in different population and age groups? (Research question 7)

We were not able to distinguish between the effect of vitamin D alone and vitamin D together with calcium on most of the health outcomes. A combination of vitamin D and calcium seems to be important in the prevention of fractures, falls, and all-cause mortality (total mortality).

#### Which is the interaction of vitamin D intake or vitamin D status with vitamin A intake or vitamin A status on health outcomes in different population and age groups? (Research question 8)

We did not find any SLRs on this topic.

#### Difference between vitamin D_2_ and D_3_ in increasing S-25-Hydroxy-vitamin D concentration

The difference between vitamin D_2_ and D_3_ was not one of our initial research questions. We, nevertheless, considered this to be an important topic that has to be included. Vitamin D_2_ and D_3_ supplementation has been reviewed in a recent SLR by Tripkovic et al. (93). Ten studies were included in the systematic review and seven studies in the meta-analysis. The doses, durations and age groups varied as well as the route of administration. The authors concluded that vitamin D_3_ is more efficacious at raising 25(OH)D than vitamin D_2_. One of the studies (94) indicated that the effect of vitamin D_2_ on S-PTH was very weak in comparison to the effect of vitamin D_3_.

### Overall discussion

Vitamin D can influence numerous biological processes in the body. In addition to the effects on bone health, it has been claimed that vitamin D contributes in the prevention of many medical conditions including CVDs, type 1 and type 2 diabetes, some types of cancer, pregnancy outcome, and infections. And indeed, there is suggestive evidence for a number of health benefits of vitamin D and for plausible biological mechanism. For example, the observation that S-25(OH)D is inversely related to some types of cancer is supported by a new reanalysis of a subgroup of participants in the large Calcium and vitamin D trial in the Women's Health Initiative (95) and associations between lowered risk for cardiovascular outcomes and higher 25(OH)D concentrations has repeatedly been observed. Similarly, higher risk for preeclampsia has been reported in pregnant women with 25(OH)D below 37.5 nmol/l and the risk for low birth weight may be lowered in at-risk pregnant women by vitamin D supplementation.

However, the SLRs we have reviewed conclude that the evidence for a protective effect of vitamin D is only conclusive concerning bone health, total mortality and the risk of falling. In addition, most intervention studies leading to these conclusions report that intervention with vitamin D combined with calcium and not vitamin D alone gives these benefits.

Currently, there is a great interest and a high research activity concerning vitamin D. Although a large number of studies, including RCTs, have been performed, there are still many unanswered questions. For example, it is unclear why combined interventions with vitamin D and calcium and not interventions with vitamin D alone have shown an effect on fracture and mortality risk. The causes for the increased risk of fracture and falling in those given a large, annual dose of vitamin D are also unclear (38), and so is the increased risk of mortality related to high concentrations of 25(OH)D reported in some studies (58, 91) Observational studies have rather consistently shown that low concentrations of 25(OH)D are related to increased risk of CVD. However, re-analyses of RCTs suggest that calcium supplementation (also with vitamin D) might increase the risk of myocardial infarction (95).

Although RCTs were emphasized in most of the SLRs, we would also point out some limitations. When interpreting the effect of doses given in RCTs, it is a challenge that the participants also receive vitamin D from other sources (diet and UVB-irradiation). In some studies, participants were also allowed to use personal supplements in addition to study medication. Basically, the RCTs give information on the effect of the difference in vitamin D exposure between the intervention group and the control group. A large difference in exposure may be difficult to obtain in RCTs. To test out the exposure of a moderate dose of vitamin D compared to very little vitamin D might therefore be difficult.

Many chronic diseases develop over many years, and it is also a challenge that RCTs in general are performed over shorter time frames. Whereas RCTs are feasible in testing out the effect and side effects of interventions with supplements, the feasibility of RCTs in establishing the relation between nutrition and disease has been debated (50).

It was difficult to establish an optimal 25(OH)D concentration or vitamin D intake based on the SLRs.

#### Other considerations

The IoM (29) also considered calcium absorption together with BMD, rickets and osteomalacia to find a totality of evidence for an optimal S-25(OH)D concentration. They found a congruence among these outcomes with no additional benefits of serum concentrations of 25(OH)D higher than 50 nmol/l.

The relationship between S-25(OH)D and S-PTH has been considered in numerous studies, and based on some of them the threshold for vitamin D sufficiency has varied between 25 and 125 nmol/l. Using S-PTH as an outcome is difficult as the variation is large and also other factors have an effect on S-PTH. Sai et al. (96) concluded in a systematic review that ‘vitamin D insufficiency should be defined as serum 25(OH)D less than 50 nmol/l as it relates to bone’.

A number of studies have shown an inverse relationship between S-25(OH)D and BMI or adiposity. Some supplementation studies, but not all, have shown a lower response in S-25(OH)D in obese persons than in normal weight subjects. Moreover, weight loss has led to an increase in S-25(OH)D in some studies. Thus, though no SLR addressed this subject, there are some indications that adiposity should be considered a determinant of S-25(OH)D-concentration (97). However, this does not suggest that there is evidence that higher intakes are needed in obese persons than in those with normal weight.

#### Limitations

We focused on SLRs and included only a few new RCTs. We were not able to perform quantitative analyses of the studies. The quality of the studies included in the SLRs varied and there was a large heterogeneity among them. All age groups were not covered and the study duration in the trials varied greatly. Different age groups were considered only in relation to bone health.

#### Study quality

Due to heterogeneity in the studies, it was difficult to interpret the results and provide single summary statements. The doses of vitamin D differed widely among the studies. Habitual vitamin D intake was seldom assessed and the methods for intake assessment varied. The assays used for the assessment of S-25(OH)D concentration varied among the studies. The study cohorts consisted mainly of Caucasians.

#### Publication bias

*Publication bias* cannot be ruled out, since relevant studies were searched in two electronic databases (MEDLINE and Swemed), and by snowballing for the last months. Unpublished or ongoing studies were not identified.

## Authors’ conclusions

### Implications for the Nordic setting

Cutaneous synthesis of vitamin D_3_ is the physiological route for vitamin D supply. Due to our geographic situation, this way of supply is turned off for about 3–5 months during the year. Dietary vitamin D is thus needed to keep vitamin D status at an acceptable level. The SLRs that we have reviewed gave insufficient evidence for an optimal 25(OH)D concentration and corresponding vitamin D intake levels in relation to most health outcomes. However, the association between vitamin D status and skeletal outcomes and the effect of vitamin D supplementation on skeletal outcomes give some information, while the role of vitamin D without calcium supplementation on fracture incidence is unclear. Moreover, studies on the effect of vitamin D supplementation and vitamin D fortification on 25(OH)D concentrations gives some information on how to achieve specific concentrations of 25(OH)D. In this respect, the heterogeneity in the results by the 25(OH)D assays is a formidable problem.

Many studies suggest that there is an increased risk for rickets in infants and children when S-25(OHD) concentration is <27.5 nmol/l. A threshold for 25(OH)D at 40–50 nmol/l has been suggested in the SLRs for the prevention of falls and fractures in the elderly. Solid evidence for an optimal S-25(OH)D concentration (or optimal intake) in children, adolescents and adults was not found in the SLRs relating to the health outcomes. However, a S-25(OH)D concentration of 50 nmol/l could be a reasonable threshold in these age groups also.

The dose-response studies relating vitamin D intake (fortification and supplementation) to S-25(OH)D suggested that an intake of 1–2.5 µg/day will increase the serum concentration by 1–2 nmol/l but this is dependent on the basal concentration with response to being greater when the basal concentration is low. Chung et al. (28) concluded that doses of 10–20 µg/day may be inadequate to ensure concentrations of 25(OH)D at or above 50 nmol/l in the great majority of individuals in the population if the relationship above was used in the calculations. Cashman et al. (32) using meta-regression analysis concluded that 50 nmol/l 9 µg vitamin D was needed on average in the age groups between 9 and 78 years in the winter. However, 23.5 µg vitamin D/day was needed to reach a serum level ≥50 nmol/l if interindividual variation was taken into account by inclusion of the 95% range in the meta-regression analysis. These values have to be treated with caution as few studies (data points) were included. Moreover, as these results are based on group data, the final interpretation is difficult. An approach with primary data from the studies would probably have given other results. In the original studies, there are confounding factors affecting vitamin D status that have not always been taken into account in the studies, for instance dietary vitamin D intake and sunlight exposure. This affects the basal 25(OH) D concentrations which again affects the dose-response. Thus, it is not easy to make extrapolations regarding the actual dose/intake and a final 25(OH)D concentration. Compliance has not been taken into account in the analyses. This is important, as a low compliance in an RCT leads to ‘falsely’ low 25(OH)D concentrations in the non-complying subjects, therefore increasing the variance in the data.

For those older than 3 years, a 50 nmol/l target for S-25(OH)D concentration would probably require an average intake of 10 µg/day, that is, 50% of a population may need more, 50% may need less than this value. Adding 2 SDs to this average intake would cover 97.5% of the population. Given that 2SD equal 5 µg/day, this would result in an intake of 15 µg/day. It should be considered that these values are based on studies conducted in the winter without any sunlight exposure. We do not have any data or evidence on the dietary requirement during the summer months with sunlight exposure. It may be presumed that less is needed during this period for most people to reach 50 nmol/l. Vitamin D is stored for months after summer in the body. However, it can be debated to what extent dietary recommendations should assume dermal synthesis during summer, as outdoor activity with light clothing may not be universal, particularly not with the frail elderly and the institutionalized.

The vitamin D requirement for the elderly has to be given special consideration. The synthesis of vitamin D in the skin may be reduced and the intestinal absorption of vitamin D may be lower than in younger persons. Thus, older people may need more vitamin D than younger persons. The dose of vitamin D (10–20 µg/day) showed that to reduce the risk of fracture and total mortality is challenging to translate directly to recommended intake of vitamin D. The participants in these studies also got vitamin D from other sources (background intake and dermal synthesis), and additional calcium was given. However, it seems reasonable to recommend a somewhat higher intake in the elderly due to the above-mentioned reasons.

There was no evidence for a different intake requirement in pregnancy and lactation compared with the general population. There is some concern about the vitamin D status in obese persons. We did not find any evidence for different recommendation among ethnic groups.

An upper tolerable level (UL) was not possible to establish based on the SLRs. There is some concern that higher S-25(OH)D concentrations is associated with an increase in mortality. Notable is that the IoM as well as the European Food Safety Agency have set the Upper Tolerable Intake for adults at 100 µg/day (29, 98).

In conclusion, if 97.5% of the population up to 75 years of age is to maintain the target 50 nmol/l concentration of 25(OH)D, the corresponding intake of vitamin D would be 15 µg/day. Higher intakes may be needed to cover this same percentage in an older population. Here, we refer to the total intake from food as well as supplements, given minimal sun exposure. Limited sunshine, however, does not reflect the situation for the majority of the Nordic population in the summertime. It should also be emphasized that there are large differences in results depending on assay methods and laboratories measuring 25(OH)D, adding to the uncertainty of determining an appropriate target concentration. Moreover, the dose response of vitamin D on serum 25(OH)D-concentrations is not well established and is dependent on the basal concentrations, sunshine exposure and dietary intake.

#### Implications for research

We have been able to identify some implications for the research:

1. The role and dose response of sunshine
2. Standardization of serum/plasma 25(OH)D assays
3. Genes regulating the 25(OH)D concentration
4. Bioavailability of vitamin D from different food sources
5. Vitamin D status and adverse effects, including mechanisms
6. Vitamin D's effects on various health outcomes
7. Vitamin D dosing (including food-based), 25(OH)D, and health outcomes.
